# A high rate of polymerization during synthesis of mouse mammary tumor virus DNA alleviates hypermutation by APOBEC3 proteins

**DOI:** 10.1371/journal.ppat.1007533

**Published:** 2019-02-15

**Authors:** Benedikt Hagen, Martin Kraase, Ivana Indikova, Stanislav Indik

**Affiliations:** Institute of Virology, University of Veterinary Medicine Vienna, Vienna, Austria; Duke University Medical Center, UNITED STATES

## Abstract

Retroviruses have evolved multiple means to counteract host restriction factors such as single-stranded DNA-specific deoxycytidine deaminases (APOBEC3s, A3s). These include exclusion of A3s from virions by an A3-unreactive nucleocapsid or expression of an A3-neutralizing protein (Vif, Bet). However, a number of retroviruses package A3s and do not encode apparent *vif*- or *bet*-like genes, yet they replicate in the presence of A3s. The mode by which they overcome deleterious restriction remains largely unknown. Here we show that the prototypic betaretrovirus, mouse mammary tumor virus (MMTV), packages similar amounts of A3s as HIV-1ΔVif, yet its proviruses carry a significantly lower level of A3-mediated deamination events than the lentivirus. The G-to-A mutation rate increases when the kinetics of reverse transcription is reduced by introducing a mutation (F120L) to the DNA polymerase domain of the MMTV reverse transcriptase (RT). A similar A3-sensitizing effect was observed when the exposure time of single-stranded DNA intermediates to A3s during reverse transcription was lengthened by reducing the dNTP concentration or by adding suboptimal concentrations of an RT inhibitor to infected cells. Thus, the MMTV RT has evolved to impede access of A3s to transiently exposed minus DNA strands during reverse transcription, thereby alleviating inhibition by A3 family members. A similar mechanism may be used by other retroviruses and retrotransposons to reduce deleterious effects of A3 proteins.

## Author summary

Retroviruses have evolved multiple means for evading host restriction factors such as APOBEC3 proteins that lethally deaminate the intermediate product of reverse transcription reaction–single-stranded cDNA. Mouse mammary tumor virus (MMTV), although it does not encode an APOBEC3-neutralizing gene product and packages APOBEC3 proteins into the cores of virions, evades accumulation of the APOBEC3-mediated G-to-A mutations. Here, we show that a point mutation in the DNA polymerase domain of MMTV reverse transcriptase (F120L), which reduced the rate of DNA synthesis, increased APOBEC3-mediated mutation levels and, in turn, sensitivity to inhibition by APOBEC3 proteins. A similar APOBEC3 sensitizing effect was detected for cell culture conditions that slow down the rate of reverse transcription reaction such as decreased dNTP levels within target cells or the presence of sub-optimal, non-lethal concentrations of reverse transcriptase inhibitors. Thus, our results support the concept that MMTV has evolved reverse transcriptase to catalyze virus DNA synthesis with a rate that alleviates APOBEC3-mediated hypermutation of the virus replication intermediates. A similar mechanism may be used by other reverse transcriptase-containing viral pathogens to escape APOBEC3 innate immunity factors.

## Introduction

Reverse transcription (RTN) is an essential step in the life cycle of all retroviruses and retrotransposons. It is catalyzed by retroviral reverse transcriptase (RT) that converts single-stranded (ss) plus-sense viral RNA genomes to double-stranded viral DNA. RTN is a vulnerable step in the retrovirus life cycle. It can be inhibited by host restriction factors including (i) TRIM5α, which destabilizes post-entry viral capsids thereby compromising timely onset/completion of RTN [1]; (ii) SAMHD1, which interferes with RTN by depleting cellular dNTP pools [2] and (iii) APOBEC3 protein family members (A3s). *A3* genes show profound copy number and amino acid variation in mammals. The mouse genome contains one A3 gene (*mA3*), whereas the human *A3* locus encodes seven A3 proteins that exhibit various degrees of antiviral potency. The most widely studied human restriction factor, A3G, interacts with nucleocapsid protein and viral RNA, thereby ensuring its packaging into viral cores, transmission to target cells and finally its presence in reverse transcription complexes. During RTN, A3G extensively deaminates deoxycytidine to deoxyuridine residues (C-to-U) in the negative strand of retroviral DNA, resulting in G-to-A hypermutation in the newly synthesized plus DNA strand [3–5]. As both human A3G and murine mA3 deaminate exclusively ssDNA intermediate products of RTN [6–8], their antiviral function can be exerted only during a finite period of time when the viral minus DNA strand remains single-stranded. This is determined by the time between synthesis of minus DNA strand, followed by degradation of the RNA template by the RT-associated RNase H activity, and synthesis of the plus DNA strand. As various regions of the minus DNA strand remain single-stranded for a different amount of time, the retroviral genomic DNA contains A3-induced mutational gradient peaking just 5’ (when considering plus strand sequence) to the polypurine tract (PPT) sequence [6, 8, 9].

Given that antiviral activity via detrimental hypermutation is limited in time, it is conceivable that differences in the kinetics of RTN among retroviruses determine their sensitivity to inhibition by A3s. Retroviral RTs are known to substantially differ in their structures and subunit composition as well as in their enzymatic properties [10]. For example, the RT of lentiviruses, including HIV-1, functions as a heterodimer composed of large and small subunits and exhibits a low processivity [10]. Conversely, RT of the prototypic betaretrovirus, mouse mammary tumor virus (MMTV) is active as a monomer and its processivity is substantially greater than that of the HIV-1 RT [11]. Although differences in the rate of DNA polymerization between retroviruses have not been extensively studied, it has been proposed that the rate of DNA synthesis correlates with the RT processivity [12, 13]. Therefore, we sought to investigate whether retroviruses with markedly distinct RT processivities differ in their sensitivity to inhibition by ssDNA-specific deoxycytidine deaminases.

MMTV, which was discovered in the 1930s as a milk-transmitted, infectious agent causing mammary tumors in adult female mice, is one of the best studied oncogenic viruses [14]. The virus is only partially sensitive to inhibition by mA3 and human A3G proteins [15, 16]. In mA3 knockout mice, MMTV replicates with slightly accelerated kinetics compared to wild-type (WT) littermates [15]. Viral particles obtained from mammary glands of MMTV-infected WT mice contain mA3 that is packaged into the cores of virions and retains its deaminase activity. However, the encapsidated mA3 does not hypermutate the MMTV genome [17]. Lack of hypermutation was also reported for MMTV produced in cells expressing human A3G. Although the producer cells expressed A3G at the levels that efficiently repressed infectivity of Vif-deficient HIV-1 (HIV-1ΔVif), only moderate levels of G-to-A mutations of the MMTV genome were observed [16]. These results suggested that MMTV has evolved a mechanism to counteract the deamination activity of A3 proteins allowing replication of the virus in the presence of the restriction factor. This mode of A3 evasion seems to be different from the mechanisms used by other retroviruses to neutralize A3 proteins, such as A3 avoidance or expression of A3-inhibiting accessory proteins (Vif, Bet) [18–22].

Here, we aimed to elucidate how MMTV evades accumulation of destructive levels of APOBEC3-induced G-to-A mutations. Direct comparison between MMTV and HIV-1ΔVif revealed that although MMTV does not encode an APOBEC3-neutralizing protein and encapsidates the same amounts of mA3 and A3G as the lentivirus, its genome contains lower levels of A3-mediated G-to-A mutations than HIV-1ΔVif. A potential explanation for the resistance to APOBEC3-induced mutagenesis could be the difference in kinetics of RTN. We tested this hypothesis by directly comparing RTs from the two viruses. We find that the MMTV RT is indeed more processive than HIV-1 RT [11] and also that it exhibits a faster rate of DNA polymerization during RTN. When the rate of DNA polymerization is reduced by mutating the F120 residue in the active center of the DNA polymerase domain of MMTV RT, the mutant virus becomes more sensitive to inhibition by mA3 and A3G and accumulates more G-to-A mutations in its genome. Similar APOBEC3-sensitizing effect can be also observed when the rate of DNA polymerization is reduced by other means including decreased concentrations of deoxyribonucleotides in the cytoplasm of infected cells (induced by treatment with hydroxyurea) or the presence of sub-optimal concentrations of an RTN inhibitor that reduces but does not abolish infectivity of MMTV. Collectively, these data provide insight into how the kinetics of RTN may be related to the sensitivity of MMTV to A3s. These findings may point towards a general mechanism used by retroviruses lacking an A3-neutralizing accessory protein to reduce their vulnerability to A3-mediated deamination: evolution of an RT that limits access of APOBEC3s to the RTN intermediate (-ssDNA).

## Results

### MMTV is less sensitive to A3-mediated restriction than HIV-1ΔVif

**Fig 1:**
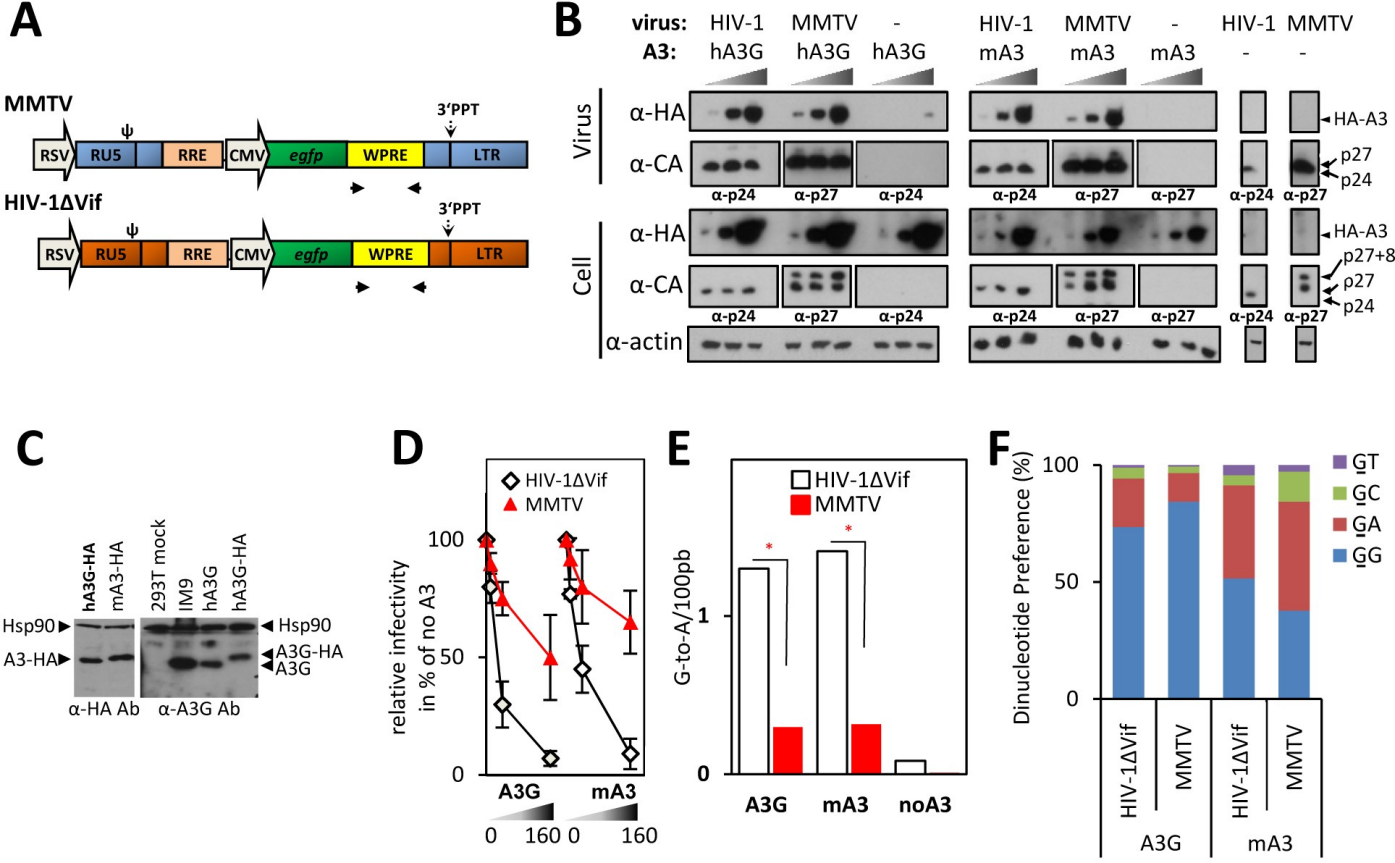
Although MMTV encapsidates the same levels of A3s as HIV-1ΔVif, it accumulates lower levels of G-to-A mutations and is less sensitive to inhibition by A3s than the lentivirus. (A) A schematic diagram of the virus expressing constructs. The RSV U3 was used for a constitutive expression of viral RNA. Both viruses carried an identical internal cassette RRE-CMV-*egfp*-WPRE; primers used for amplification of the WPRE region positioned in the vicinity to 3’PPT are displayed as black arrows. (B) Immunoblots of cellular and viral lysates. MMTV and HIV-1ΔVif viruses, produced from 293T cells transfected with increasing amounts of HA-tagged A3 expression construct (10 ng, 40 ng, 160 ng; a right black triangle), were collected and concentrated by ultracentrifugation. An *egfp*-specific TaqMan RT-qPCR was used to confirm equivalent virus loading for semi-quantitative immunoblots. The lysates from virus-producing cells were prepared and 10 μg of total cellular proteins were used for the immunoblot. Antisera specific for the HA epitope present on the A3 proteins (α-HA), the HIV-1 capsid (α-CA, α-p24) or the MMTV capsid protein (α-CA, α-p27) and actin were used. (C) Immunoblot analysis of cellular lysates comparing protein levels of ectopically expressed A3s vs. endogenous A3G. The left panel shows a representative example of a comparison of the A3-HA expression levels in virus-producing cells transfected with 40 ng of the A3G or mA3 expression plasmid (detected by anti-HA antibody). No obvious differences in the A3 expression levels between cells producing HIV-1ΔVif and MMTV were detected (see Fig 1A). The right panel shows the expression levels of HA-tagged and untagged A3G in the transfected 293T cells (40 ng of plasmid DNA) and in a lymphoblastoid cell line producing endogenous A3G (IM9) detected by the anti-A3G antibody. Anti-Hsp90 antibody was used to control equivalent loading. (D) An A3 dose-response analysis of inhibition of the MMTV and HIV-1ΔVif infectivity. The *egfp*–containing viruses were produced in 293T cells expressing increasing levels of A3 proteins (transfected with 0 ng, 10 ng, 40 ng, 160 ng of plasmids). At 42 h post-transfection, virions were collected and used to infect naïve 293T cells. Detection of EGFP-positive cells was performed 48 h post-infection. Data are presented relative to a control virus prepared in the absence of A3s. The average of three independent experiments with standard deviation is shown. Cell lysates and aliquots of viruses from one of the three replicates were used for the immunoblot shown in Fig 1B. (E) Quantification of the A3G- and mA3-mediated G-to-A transitions in the MMTV and HIV-1ΔVif genomes from the infectivity experiment shown in Fig 1D. The DNA from cells infected with viruses produced in the absence of A3s or in the presence of A3G or mA3 (40 ng of plasmid DNA) was used as a template for amplification of an ~500 bp-long fragment derived from the WPRE region present in both viruses. The frequency of G-to-A mutations in the amplified DNA was determined and differences between viruses tested by a χ^2^ test (* P<10^−5^; n_MMTV/hA3G_ = 296 sequences; n_MMTV/mA3_ = 232 sequences; n_MMTV/noA3_ = 185 sequences; n_HIV1/hA3G_ = 213 sequences; n_HIV1/mA3_ = 452 sequences; n_HIV1/noA3_ = 179 sequences). (F) A histogram summarizing the frequency of G-to-A mutations detected within each dinucleotide context (data from Fig 1E). The mutated G is underlined.

Previous studies suggested that in the presence of mA3 and A3G MMTV accumulates less G-to-A mutations and is inhibited to a lesser extent than other retroviruses, including Vif-deficient HIV-1 (HIV-1ΔVif)[15, 16, 23]. To test the ability of MMTV to withstand restriction by various A3 proteins, we performed a dose-response analysis in a single-round infection assay using an enhanced green fluorescent protein (GFP)-expressing MMTV [24]. Reduction of the MMTV infectivity was compared to the inhibition detected for a control recombinant *gfp* gene-containing virus derived from HIV-1ΔVif, which is known to be potently inhibited by A3s. The viruses were produced in 293T cells by co-transfecting a packaging plasmid expressing the HIV-1 or MMTV Gag and Pol proteins together with: an HIV-1- or MMTV-based reporter vector (Fig 1A); a construct expressing the VSV-G envelope protein; a plasmid encoding an RNA export-promoting factor; and with one of two A3 expression construct. We used HA-tagged versions of a potent mouse A3 allelic variant from C57BL/6 lacking exon five (mA3) and a human A3 protein (A3G), the two A3s used in previous studies with MMTV [15, 16, 23, 25].

Initial experiments revealed that transfection of equal amounts of HIV-1 and MMTV packaging plasmids (0.8 μg) resulted in more efficient production of lentiviral compared to betaretroviral particles as determined by an RT-qPCR with *egfp*-specific primers (both viruses contain *egfp* gene). We anticipated that unequal virus production may affect the comparison of the two viruses with respect to their sensitivity to A3. Therefore, we titrated the HIV-1 packaging construct to decrease the amount of HIV-1 virions to the levels found in the MMTV preparations (S1A Fig). Transfection of 0.2 μg and 0.8 μg of the lentiviral and MMTV packaging plasmid, respectively, led to the generation of an equal amount of virions in both preparations (S1B Fig); hence these amounts were used for further studies.

As shown in Fig 1B and 1C, A3G and mA3 proteins were efficiently expressed in the virus-producing cells and their expression level was similar to the A3G levels in a human B-lymphoblastoid cell line, IM9 (Fig 1C, cells transfected with 40 ng of A3 expression constructs). Further, dose-response analysis revealed that although the mouse and human A3s attenuated the infectivity of both viruses in dose-dependent manners, their antiviral potency against the two viruses markedly differed. Specifically, the anti-HIV-1ΔVif effect of the A3G was greater than the anti-viral effect detected for MMTV (Fig 1D). For example, transfection of 40 ng of A3G plasmid to HIV-1ΔVif-producers reduced the HIV-1ΔVif infectivity by approximately fourfold. In contrast, the infectivity of MMTV was reduced by only ~1.2 fold. A similar difference in sensitivity to inhibition between the two viruses was also detected for the mA3 (Fig 1D). Thus, this result supports the concept that MMTV is not as potently inhibited as HIV-1ΔVif and suggests that the betaretrovirus has evolved a broadly acting anti-A3 mechanism.

### Inhibition of infectivity is accompanied by G-to-A mutations

Inhibition of retroviral infectivity by A3 proteins usually correlates with an extensive deamination of deoxycytidine in the nascent reverse transcripts. However, previous results with MMTV suggested that the newly synthesized DNA is deaminated to a lesser extent, although the virion-associated mA3 retains its deamination capacity [16, 17, 23]. To test whether the lower suppression of MMTV infectivity correlates with a lower frequency of G-to-A mutations in MMTV DNA compared to HIV-1ΔVif DNA, we determined the rate of G-to-A editing in reverse transcripts of both viruses. We took advantage of the fact that both vectors carried an identical 2.9 kb-long region spanning the internal part of the vectors (RRE-*egfp-*WPRE). The use of such vectors eliminates the possibility that the differences in deamination frequency could be attributed to differences in RNA structure, which may cause pausing of RT at sites with a complex RNA folding, resulting in a more frequent RNA cleavage and a higher rate of deamination at these sites. We inspected proviruses generated by virions produced in cells either lacking A3s or expressing one of the A3s, mA3 or A3G (Fig 1D). A ~500 bp-long portion of the viral reverse transcripts derived from the woodchuck hepatitis virus post-transcriptional regulatory element (WPRE) present in both viral DNAs in the vicinity of the polypurine tract (PPT) was amplified from infected cells and subjected to high throughput sequencing. A comparison of the sequences recovered from infected cells revealed a significantly higher rate of G-to-A editing for HIV-1ΔVif than for MMTV, regardless of whether the viruses were produced in cells expressing mA3 or A3G (Fig 1E). The level of mutations observed in HIV-1ΔVif DNA in the presence of A3G (1.3/100bp) and mA3 (1.4/100bp) was consistent with previous reports [26, 27]. The MMTV DNA made in the presence of A3G and mA3 showed a mutation frequency of 0.3/100bp and 0.32/100bp, respectively. The four-fold difference in deamination rates between MMTV and HIV-1ΔVif reverse transcripts correlated with approximately four-fold less potent inhibition of the MMTV infectivity compared with that of HIV-1ΔVif (Fig 1). Consistent with previous reports the A3G-induced mutations were preferentially detected in the GG dinucleotide context, whereas the mouse A3-induced changes were more frequently found in the GA dinucleotide-containing substrates (Fig 1F, S2 Fig). Thus, these results suggest that the modest deamination of MMTV reverse transcripts underlies relative resistance of MMTV to A3s.

### MMTV efficiently encapsidates A3 proteins

To inhibit infectivity, A3 proteins have to be packaged into the cores of virions, enter target cells within viral cores and exert their inhibitory effect during reverse transcription. Previous reports showed that MMTV packages A3s into the cores [15, 17, 23], however, the levels of packaged proteins was not compared with other retroviruses. Different amounts of packaged A3s may influence the sensitivity of viruses to inhibition by A3s. Therefore, we tested whether the less potent inhibition of MMTV infectivity relative to HIV-1ΔVif results from a less efficient packaging of A3s by MMTV. We produced both viruses in the presence of A3G or mA3 proteins and examined their amounts in the MMTV and HIV-1 virus particles. Equivalent virus levels (verified by RT-qPCR) were used as an input for Western blot analysis. As demonstrated in Fig 1B, MMTV incorporated approximately the same amounts of A3G as HIV-1ΔVif. A similar result was obtained with mA3, which was also efficiently packaged into both betaretroviral and lentiviral virions. Thus, we conclude that poor restriction of MMTV infectivity cannot be explained by a less efficient incorporation of A3 proteins into MMTV particles.

### MMTV does not encode a factor inhibiting the deaminase activity of A3s

**Fig 2:**
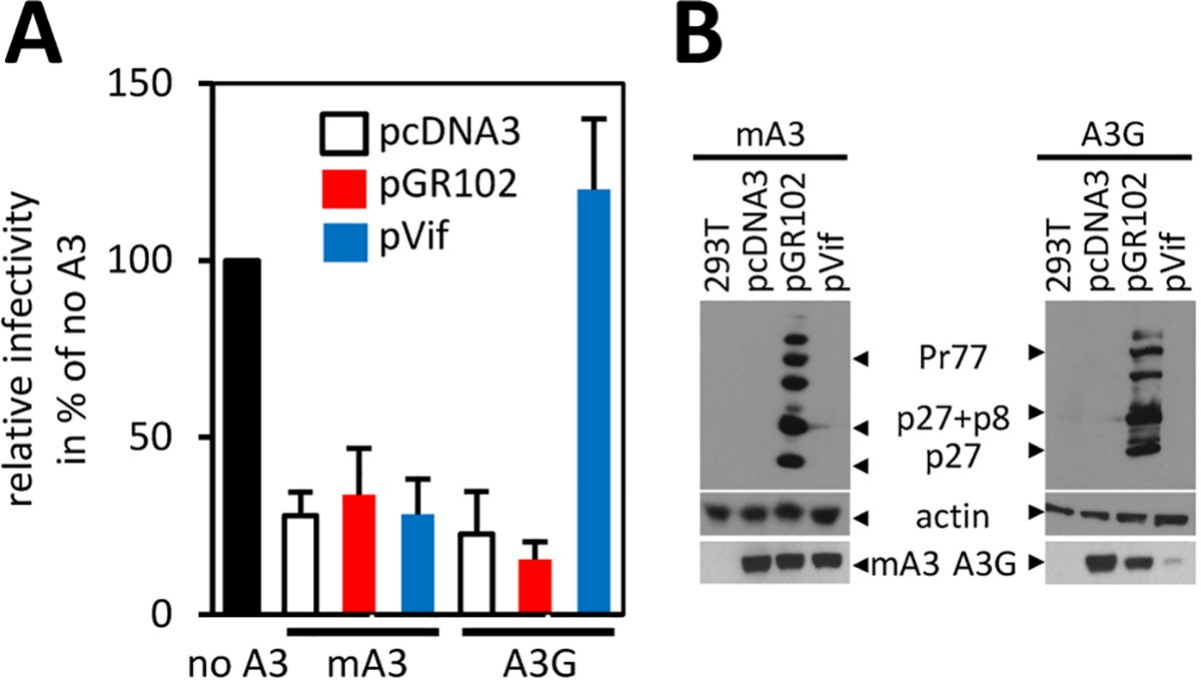
MMTV does not encode a factor antagonizing the A3 proteins. (A) For a trans-complementation assay HIV-1ΔVif vector was produced in the absence (no A3s) or presence of either A3G or mA3 (40 ng). A complete MMTV molecular clone (pGR102), a Vif-expression construct (used as a positive control) or an “empty” pcDNA3 plasmid (used as a negative control) were provided in trans. The pcDNA3 was used to adjust DNA amount to 3 μg. Data are shown relative to a control virus prepared in the absence of A3G. Mean values with standard deviation from three experiments are shown. (B) Immunoblots of cell lysates (10 μg) from transfected cells probed with α-p27 or α-actin antibody are shown. The expression of A3G and mA3 following transfection with the trans-complementing expression factor was monitored using α-HA antibody.

Retroviruses are known to express several gene products to antagonize A3s. These include Vif of HIV-1, which prevents incorporation of A3G into virions by inducing A3G proteasomal degradation [28–32], or Bet protein of foamy viruses that sequesters A3s away from the virus assembly site, thereby preventing their incorporation into virions [20, 21, 33]. To investigate whether MMTV encodes a factor neutralizing the deaminase activity of A3s we established a trans-complementation assay aiming to rescue the infectivity of HIV-1ΔVif produced in the presence of A3G and mA3, respectively. The virus was generated in mA3- or A3G-expressing 293T cells together with either HIV-1 Vif (as a positive control), MMTV gene products expressed from a complete infectious molecular clone of MMTV (pGR102 [34]) or no trans-activating factor (pcDNA3). We anticipated that if MMTV produces a factor that interacts with A3s and neutralizes their deaminase activity, the presence of the factor should enhance the infectivity of HIV-1ΔVif produced in cells expressing the innate immunity protein. In accordance with previous data [30], the presence of Vif reduced the A3G expression levels in cells and resulted in an enhanced infectivity of HIV-1ΔVif compared to the virus produced in the absence of trans-complementing factor (pcDNA3). This effect was specific to A3G as the levels of mA3 protein and the HIV-1ΔVif infectivity remained unchanged when Vif protein was expressed in mA3-producing cells (Fig 2A and 2B). Further, as also shown in Fig 2, complementation in *trans* between HIV-1ΔVif and MMTV did not restore the infectivity of HIV-1ΔVif produced in the presence of either A3G or mA3, even though the MMTV gene products were readily detectable in the transfected cells. These results suggest that the betaretrovirus does not encode a gene product with an A3-counteraction activity.

### The MMTV RT exhibits a faster rate of DNA polymerization during RTN than the HIV-1 RT

**Fig 3:**
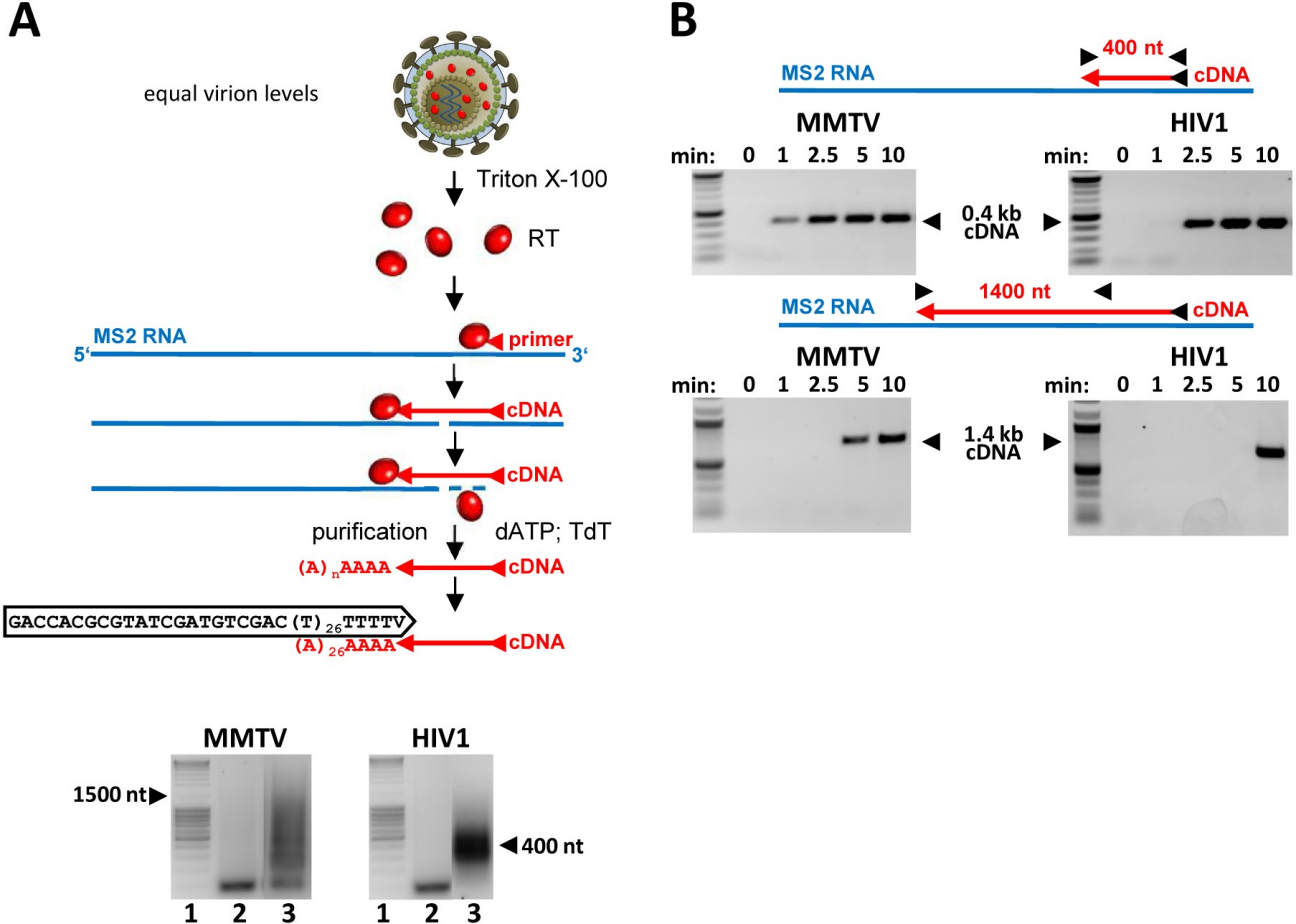
MMTV RT is more processive and faster than HIV-1 RT. RT enzymes were extracted from preparations containing equal virion levels (quantified by RTqPCR and verified by physical particle counting (EM, S3 Fig). (A) To determine RTs processivity an MS2 cDNA was synthesized in the presence of an excess of the template and a trap to limit re-association of RTs with cDNAs. The cDNA products were A-tailed and amplified using anchor- and MS2-specific primers. A schematic diagram of the method is shown in the upper panel. The length distribution of the cDNA products was analyzed on 1.5% agarose gels (lower panel); lane 1: MassRuler DNA ladder (Fermentas), lane 2: extension terminated after 0 min, lane 3: extension terminated after 15 min. The assay was repeated three times with similar outcome. (B) Kinetics of MS2 cDNA synthesis was assayed in the absence of the trap and with a limited amount of the MS2 RNA/primer template. The cDNA polymerization was terminated after the various amount of time and the presence of 0.4 kb- or 1.4 kb-long cDNA determined by PCR with MS2-specific primers. PCR products were analyzed on agarose gels (marker: 2-Log DNA ladder (NEB)). The Fig shows a representative example of three assays.

Next, we hypothesized that the poor deamination of the MMTV RTN intermediates can be attributed to an intrinsic property of the MMTV RT. During RTN the deaminase activity of A3s is restricted to deoxycytidines on the (-)ss DNA intermediate [6, 7]. The minus DNA strand is available only for a finite period of time determined by the time span between the minus DNA strand synthesis (with concomitant degradation of the viral RNA template) and the generation of the plus DNA complement. Thus, the kinetics of RTN defines the amount of time for which the minus DNA strand is vulnerable to A3s-mediated deamination. Thus, it seems reasonable to assume that differences in the rate of DNA polymerization between viruses may contribute to differences in the magnitude of inhibition by A3s. To analyze differences between the HIV-1 RT and MMTV RT we first conducted a processivity assay with the two RTs. The processivity is defined as the number of deoxynucleotides incorporated into nascent DNA before the enzyme dissociates from the template. The DNA polymerization reaction is performed with an excess of a trap (heterologous DNA:DNA primer complex) ensuring that only a single polymerization event is allowed. Virus stocks were produced in the absence of A3s as described for Fig 1 and concentrated by ultracentrifugation. The RT enzyme was released from the virions by a detergent treatment and the virion-extracted enzymes were incubated with a pre-formed MS2 RNA template:MS2 DNA primer complex before addition of a saturating concentration of dNTPs (200 μM) and the trap. Next, the 3’end of synthesized cDNA was dA-tailed using a terminal transferase and amplified by a PCR with dT-anchor- and MS2-specific primers. Length distribution of the amplicons was analyzed by agarose gel electrophoresis. Consistent with previously published data [11], we observed a marked difference between the two RTs. Whereas the MMTV RT was capable of producing cDNA with a maximal length of ~1.5 kb, the HIV-1 RT extended the primer only up to ~0.4 kb (Fig 3A).

Based on the observed difference in processivity it is tempting to speculate that the two viruses differ in the rate of DNA synthesis. To directly investigate the kinetics of RTN catalyzed by the MMTV RT and HIV-1 RT we set up a DNA synthesis rate assay. The MS2 cDNA was produced by RT extracted from equal amounts of virions (equal RNA genome equivalents) for each virus. For this assay electron microscopy was also used to verify equivalent amounts of virions in the preparations (S3 Fig). The advantage of the method is that it quantifies RT activity per a virion rather than the activity of equimolar amounts of recombinant enzymes. The MS2 cDNA synthesis was carried out in the absence of the trap (to enable multiple rounds of RT binding to the nascent DNA) and with 50 μM dNTP. The reactions were stopped at various time points following initiation (0, 1, 2.5, 5 and 10 min) and the presence of cDNA was analyzed by PCR with MS2-specific primer pairs designed to amplify a ~0.4 kb- and 1.4 kb-long MS2 cDNA. As shown in Fig 3B (upper panel) a low amount of the 0.4 kb MS2 cDNA synthesized by the MMTV RT was detectable already 1 min after the initiation of RTN, suggesting that some cDNA synthesis was completed in less than 1 min. The time required by the HIV-1 RT to synthesize the MS2 cDNA was consistently longer compared to that needed by MMTV RT. Approximately 2.5 min were required for the extension of the 400 nt from the RTN initiation site to the PCR primer. The difference in the rate of DNA synthesis was more pronounced when the production of the longer cDNA was analyzed. While it took the HIV-1 RT 10 min to synthesize the 1.4 kb-long cDNA, the MMTV RT needed only approximately 5 min to complete the cDNA synthesis (Fig 3B; lower panel). Collectively, the presented evidence demonstrates that MMTV RT has a higher processivity and a faster rate of DNA synthesis than HIV-1 RT, further supporting the hypothesis that variations in the DNA polymerization rate during RTN are responsible for differences in the frequency of A3s-mediated mutagenesis.

### F120L mutant is slower in cDNA synthesis than WT virus and exhibits an increased sensitivity to inhibition by A3s

**Fig 4:**
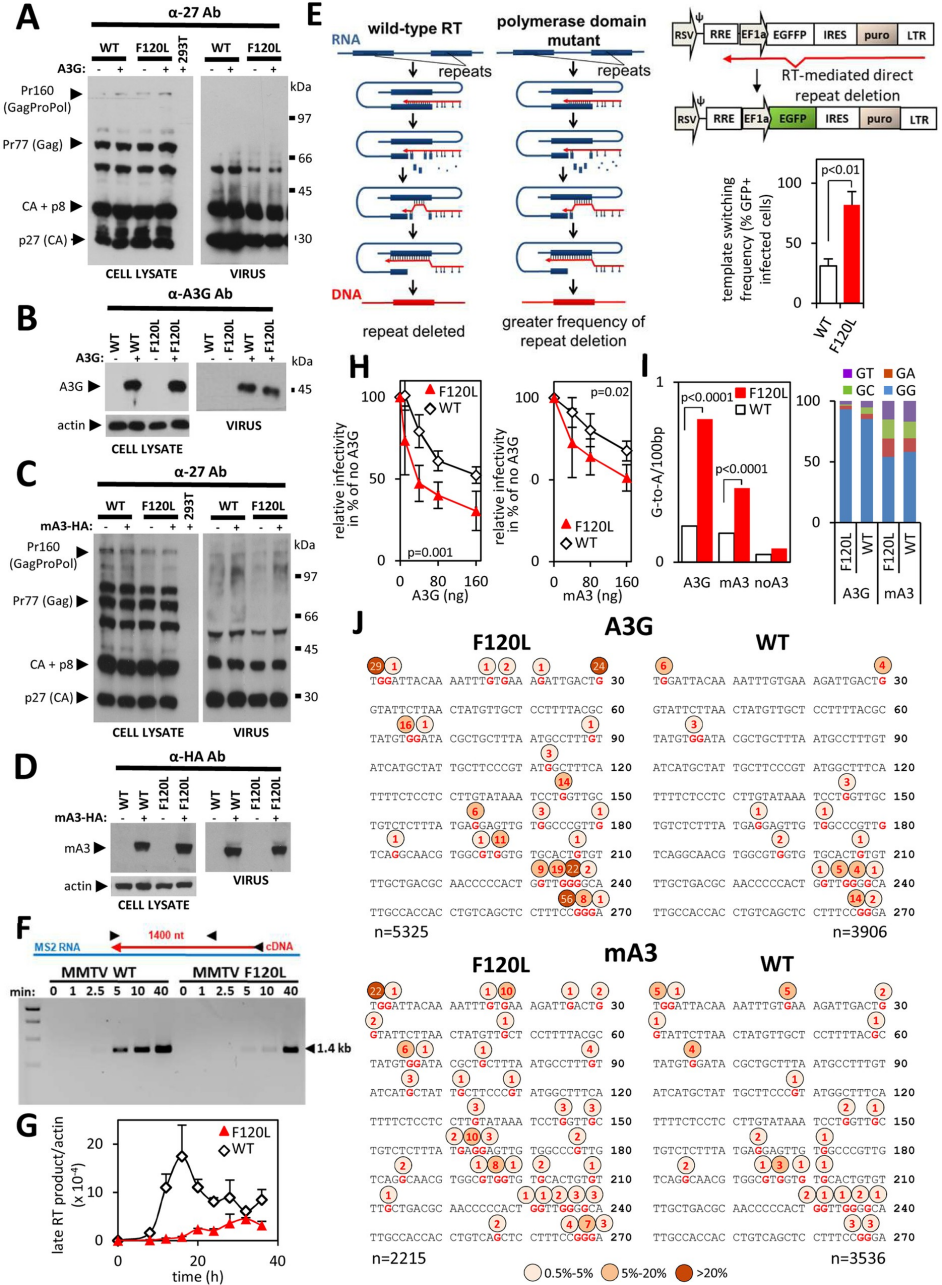
The F120L mutation, which does not affect A3s packaging, reduces the rate of DNA synthesis and sensitizes MMTV to inhibition by A3s. (A—D) Detection of MMTV-specific proteins using anti-p27 (CA) antibody in cellular and viral lysates. For cellular lysates, 10 μg of total proteins obtained from MMTV WT or MMTV F120L virus-producing cells were used. Virus lysates were prepared from 100-fold concentrated virus preparations obtained from the supernatant of the MMTV WT or MMTV F120L virus-producing cells. The blots show unchanged expression levels and processing of capsid protein precursors following the introduction of the F120L mutation to the *pol* gene (A and C; left panel). Both, MMTV WT and MMTV F120L virions were also efficiently released from transfected cells (A and C; right panel). (B and D) Immunoblot detection of A3G and mA3 in the same lysates as shown in (A) and (C), respectively. An anti-actin antibody was used to show equivalent loading with cellular lysates. (A—D) One of four independent virus preparations, which were used for Fig 4H (right graph), I and J is shown. (E) A recombination assay. Direct repeats (thick blue line) are frequently deleted from retroviral genomes. Based on a dynamic copy choice model the deletion occurs when nascent minus DNA strand (red line) anneals to complementary RNA strand (blue line) situated upstream from the polymerizing reverse transcriptase (RT). The frequency of the deletion correlates with the length of the single-stranded (ss) DNA generated by RT. The longer ssDNA regions are generated with an RT exhibiting reduced activity of the DNA polymerase domain relative to the RNase H domain (polymerase domain mutants). Hindered DNA polymerization allows more efficient RNA degradation resulting in longer (-)ssDNA segments. The longer (-)ssDNA regions facilitate hydrogen bonding with the acceptor RNA template and in turn, enhance the recombination rate. The same mechanism applies to an *egffp* reporter gene carrying a direct repeat (f) in the central part of the *egfp* gene. Hydrogen bonding between nascent (-)ssDNA and viral RNA facilitates template switching and leads to deletion of the repeat. Reconstitution of the *egfp* gene results in the expression of functional EGFP that can be detected by UV microscopy and flow cytometry (right panel). Template switching frequency was determined for the MMTV WT RT and the MMTV F120L RT mutant. Differences between viruses were tested using Student’s two-tailed t-test (right panel) in GraphPad Prism. Results shown represent mean values with standard deviations from three experiments. A low MOI (<0.01) was used to ensure a single infection event per cell. A puromycin selection after infection ensures that only the infected cells are used for quantification of EGFP-expressing cells by flow cytometry. (F) The difference in the rate of DNA synthesis between the MMTV WT RT and MMTV F120L RT was analyzed using RTs liberated from virions, which were normalized to equal viral RNA levels (verified by EM, S3 Fig). The MS2 cDNA was synthesized and detected as described for Fig 3B. (marker: 1 kb DNA ladder, NEB). A representative Fig from three independent experiments is shown. (G) Accumulation of the late RT products in infected cells. DNA was extracted from 293T cells infected with MMTV WT or MMTV F120L virus preparations. DNA (100 ng) obtained at the indicated time points (0 h– 40 h) were subjected to a qPCR specific for the late MMTV RT products. Values were normalized to actin DNA levels. (H) mA3- and A3G-mediated inhibition of infectivity. Dose-response analysis of the inhibition of virus infectivity was performed as described in Fig 1D. Five independent virus preparations in five dose response infection experiments were performed for experiments with the mA3 (left). Four virus preparations were performed for the A3G tests (right). A two-way ANOVA in GraphPad Prism was used to analyze data and we found that the mutation significantly affects response to mA3 and A3G (I) Frequency of G-to-A editing found in the WPRE portion of the MMTV WT and MMTV F120L proviruses. The genomic DNA was extracted from cells infected with viruses produced in the absence (no A3G) or presence of A3G or mA3 (40 ng of A3 expression plasmid). A χ^2^ test performed in GraphPad Prism was used to analyze the differences in the frequency of deamination between viruses. (J) A G-to-A editing pattern detected in the plus DNA strands of the MMTV WT and MMTV F120L proviruses. Percent of mutated Gs at individual nt positions are depicted above the 270 bp consensus sequence.

To further investigate whether the faster rate of DNA synthesis catalyzed by the MMTV RT relative to the HIV-1 RT is responsible for the lower frequency of A3-mediated mutagenesis, we generated MMTV RT mutants carrying amino-acid substitutions in the dNTP binding pocket of the enzyme. We hypothesized that, analogously to HIV-1 RT mutants [35, 36], reduced dNTP incorporation kinetics reduces the rate of DNA synthesis. As a result of the kinetic interference in DNA synthesis the amount of time required for minus DNA strand synthesis lengthens resulting in a greater frequency of A3-mediated editing of the nascent DNA chain. We targeted the phenylalanine-119 of the MMTV RT that is homologous with Y-115 in HIV-1 RT and F-155 in MLV RT, and is located at the dNTP-binding site (S4A Fig)[37, 38]. Previous work showed that an F119V RT mutant exhibited a reduced dNTP binding and a lower processivity compared with the wild-type (WT) RT [37]. However, this mutation had a deleterious effect on the MMTV infectivity that precluded its usage in A3 sensitivity studies. Therefore, we generated a series of mutants carrying either another amino acid substitution at position 119 or a mutation at another residue homologous with the amino acid in HIV-1 RT interacting with incoming dNTPs (K65, Q151, F116) [38, 39]. Whereas infectivity of mutants F119L, F119W, F119P, F119Y, F120W, F120P, F120Y, F120A, D117A, D117E, Q155N and K70R was greatly reduced, a mutant carrying leucine instead of phenylalanine at position 120 retained approximately 20% of the WT virus infectivity (S4B Fig). The mutation did not have a negative effect on the expression of viral proteins nor on A3 packaging into virions and the mutant viral particles were also efficiently released from the virus-producing cells (Fig 4A–4D). We then tested whether the F120L mutation decreased the rate of DNA elongation. First, we compared the mutant with the WT virus in a direct repeat deletion assay that quantifies the frequency of template switching during RTN. Previous reports showed a correlation between the rate of polymerization and the template switching frequency. Genetic alterations of HIV-1 RT that interfered with dNTP binding, such as Q151N, V148I, K65R, Y115F, and F116Y, impeded RT elongation and promoted template switching [35, 40]. Therefore, we postulated that if the F120L mutation delays minus DNA strand synthesis, we should detect an increased rate of template switching in cells infected with the mutant compared with those infected with WT virus. As described before for other retroviruses [40, 41] we generated an MMTV reporter vector with a modified *egfp* gene (*egffp*) containing two directly repeated fragments (denoted as “f”; 269 bp) separated by a 24-bp linker. Deletion of the repeat (f), which is mediated by RT and facilitated by base pairing between the synthesized minus DNA strand and a complementary template RNA region upstream of the polymerizing RT, results in reconstitution of an *egfp* gene and expression of a fluorescent EGFP protein in infected cells. As the amount of time needed for the expression of the 269 bp long repeat region defines the likelihood of hydrogen bonding between the acceptor RNA template and donor cDNA, delayed DNA synthesis increases the frequency of template switching. Using this assay, we found that the F120L mutant recombines significantly more frequently than the WT virus. This was evidenced by two to three-fold increased frequency of *gfp* reconstitution detected for the F120L mutant relative to WT virus (Fig 4E). The result suggests that the F120L mutation in the active site of the DNA polymerase domain of MMTV RT decreased the rate of DNA elongation during RTN.

To provide further evidence for kinetic interference of the mutation on DNA synthesis, we measured the rate of the MS2 cDNA elongation catalyzed by RTs extracted from the same amount of F120L and WT virions as described above. We focused on the longer cDNA product as the difference was more pronounced than for the shorter cDNA. Using the primer pair (1252R and 512F) designed to detect the 1.4 kb-long MS2 cDNA we observed that the RT from WT virus needed ~5 min for progression of RTN from the initiation site to the 512F primer. In contrast, RTN products generated by the F120L RT accumulated with a slower kinetics than the cDNA elongated by WT RT. More than 10 min was required by the mutant RT for completion of elongation of the 1.4 kb cDNA (Fig 4F).

Next, we determined the kinetics of accumulation of MMTV cDNA in infected cells. We performed this analysis by measuring the amount of time required for synthesis of the late RTN products with an MMTV-specific primer pair designed in the U3 (1176F) and the 5’untranslated region (1495R). As viral RNA is expressed in transfected, virus-producing cells from a hybrid 5’LTR containing the U3 derived from Rous Sarcoma Virus and the R-U5 regions derived from MMTV (Fig 1A), an authentic MMTV 5’LTR that allows amplification of a 321 bp-long fragment of viral DNA is formed after the plus-strand DNA transfer [24]. As shown in Fig 4G, synthesis of the late WT and F120L RT products peaked at 16 h and 32 h post-infection, respectively. Thus, this analysis confirmed the *in vitro* results obtained with the MS2 RNA and established that the mutation delays the authentic MMTV DNA synthesis in cells. Of note, we also observed that although we used the same amount of viral particles for infection (*egfp* gene levels normalized), an approximately four-fold lower amount of F120L DNA compared with WT DNA was produced. Mechanistic nature underlying the defective synthesis of the late viral DNA products in the cytoplasm of infected cells is not known. We are favoring the possibility that the delayed completion of DNA synthesis may allow recognition of viral DNA as “non-self” followed by its degradation by intrinsic cellular restriction factors. Collectively, the in vitro MS2 cDNA synthesis assay and the authentic MMTV late RT synthesis assays showed a slower rate of DNA synthesis for the virus containing mutated RT.

Next, we tested whether the F120L mutation has an effect on the sensitivity to inhibition by A3s. Dose-response analyses presented in Fig 4H showed that both A3G and mA3 more effectively inhibit the F120L mutant compared to the WT virus. For example, the A3G expression level, resulting from transfection of 40 ng of A3G plasmid to virus-producing cells (which is similar to the endogenous A3G level expressed in IM9 lymphocytes (Fig 1C)), inhibited the F120L infectivity approximately three-fold. In contrast, the infectivity of wild-type virus was reduced by only <1.3 fold (Fig 4H, left). Similar results were obtained with viruses produced in the presence of mA3 (Fig 4H, right). Next, we recovered the newly synthesized proviral DNA from infected cells and subjected it to high throughput sequencing. As shown in Fig 4I and 4J, we observed a significantly higher frequency of G-to-A editing in the F120L proviral genomes than in the wild-type virus (0.77 vs 0.18 / 0.1kb). Whereas G-to-A transitions in sequences derived from the WT virus were distributed over 14 guanosine residues, sequences derived from the F120L mutant carried G-to-A mutations at 25 guanosine positions of the 270 nt-long sequenced region. Furthermore, of the 14 guanosine positions mutated in both proviruses all were mutated in the F120L proviruses with a greater frequency than in the WT viruses (Fig 4J, upper panel, S5 Fig). As expected the vast majority (93% for F120L and 85% for WT) of the mutations caused by A3G were in GG dinucleotide context (Fig 4I, right). Further, similar to A3G, analysis of sequences derived from viruses containing mA3 revealed that the mutation in the active center of RT predisposed MMTV to the more frequent accumulation of G-to-A mutations (0.41 vs 0.16 / 0.1 kb). Although the overall mutation frequency was lower compared to A3G, more guanosine positions in the analyzed region were mutated (38 for F120L and 24 for WT), suggesting that mA3 is less strict in the selection of substrate than A3G (Fig 4J, lower panel; S5 Fig). This was also reflected in the dinucleotide preference of mA3, which, when compared to A3G, showed a greater deamination frequency of GT, GA or GC substrates (Fig 4I, right).

Taken together, these results demonstrate that the F120L mutation in RT renders MMTV more susceptible to inhibition by A3s and that this phenotype results from elevated G-to-A editing frequency of the mutant.

### Decreased levels of dNTPs and suboptimal concentrations of AZT in infected cells enhance the sensitivity of MMTV to A3s

**Fig 5:**
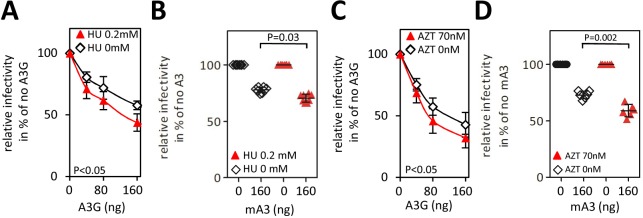
Reduction of intracellular dNTP concentrations and the presence of an RT inhibitor sensitize MMTV to A3s. (A and B) Intracellular dNTP concentrations affect the sensitivity of MMTV to A3s. Target cells were treated (4 h before infection) with a drug inhibiting ribonucleotide reductase, hydroxyurea (HU), before they were challenged with virus stocks prepared in cells expressing increasing amounts of A3G (40, 80 and 160 ng) and mA3 (160 ng), respectively. The HU treatment continued for an additional 20 h. A two-way ANOVA was used to analyze data obtained with A3G from three independent infection experiments. An unpaired two-tailed t-test was used to analyze data with mA3 obtained from six infection replicates. (C and D) Sub-optimal concentrations of an RT inhibitor, AZT, sensitize MMTV to inhibition with A3G or mA3. The IC_50_ concentration of AZT (70 nM), was added to target cells together with the virus inoculum. The virus was removed three hours after infection and a culture medium supplemented with AZT (70 nM) was added to the cells. A two-way ANOVA (C) or unpaired two-tailed t-test (D) was used to analyze data from three (C) or six (D) independent infection experiments. (A–D) Detection of GFP^+^ cells was performed as described for Fig 1D.

To further test whether the rate of DNA synthesis influences the sensitivity of MMTV to A3s we analyzed the A3-mediated inhibition in cells grown in the presence or absence of an inhibitor of cellular ribonucleotide reductase, hydroxyurea (HU). We hypothesized that a reduction of intracellular dNTP levels resulting from HU treatment [42] reduces the rate of reverse transcription [43, 44]. This leads to a prolonged exposure of the minus DNA strand to A3s and more efficient neutralization of MMTV infectivity compared with the infection of untreated cells. To test this hypothesis, 293T target cells were treated with 0.2 mM HU 4 h prior to infection, 3 h during infection and 17 h post-infection [40] resulting in a reduction of intracellular dNTPs levels and a reversible change of cellular morphology (S6 Fig). Furthermore, the treatment delayed the onset of eGFP expression following infection, indicating that the duration of reverse transcription was prolonged as anticipated (S6B Fig). The inhibition of infectivity of MMTV produced in the presence of increasing amounts of A3G was analyzed as described for Fig 1D and Fig 4H. The data presented in Fig 5A show that the presence of HU sensitized MMTV to inhibition by A3G as evidenced by a steeper decline of the virus infectivity in the presence of HU. Further, MMTV was also more sensitive to inhibition by mA3 as evidenced by significantly lower virus infectivity in target cells that were exposed to HU compared to unexposed cells (Fig 5B).

Next, we tested whether other means of inhibition of the kinetics of reverse transcription, such as the presence of an inhibitor of RT, affects the sensitivity of MMTV to inhibition by A3s. As shown in Fig 5C and 5D, similar infectivity profiles as those seen with HU-treated vs. HU-untreated cells were detected when differences in infectivity in the absence or presence of an inhibitor of RT (3’-azido-3’deoxythymidine, AZT) were followed (Fig 5C and 5D). For this analysis, we worked on the presumption that the nucleoside RT inhibitors including AZT act as chain terminators following their incorporation to the DNA during RTN. However, they may be, under suboptimal non-lethal concentrations, excised from the 3’end of nascent DNA by a pyrophosphorolysis allowing continuation of the DNA strand elongation [45]. We assumed that the termination of elongation followed by pyrophosphorolysis delays DNA synthesis allowing a more potent inhibition by A3G. To perform this analysis we infected cells with either no drug or with a suboptimal concentration of AZT (70nM), which reduced the infectivity of MMTV by ~ two-fold, and tested whether the treatment has an effect on the sensitivity of MMTV to A3G. We found that the presence of AZT resulted in a significant increase of sensitivity to inhibition by A3s (Fig 5C and 5D) further confirming our the concept that the rate of RTN is an important determinant of the sensitivity of MMTV to A3s.

## Discussion

**Fig 6:**
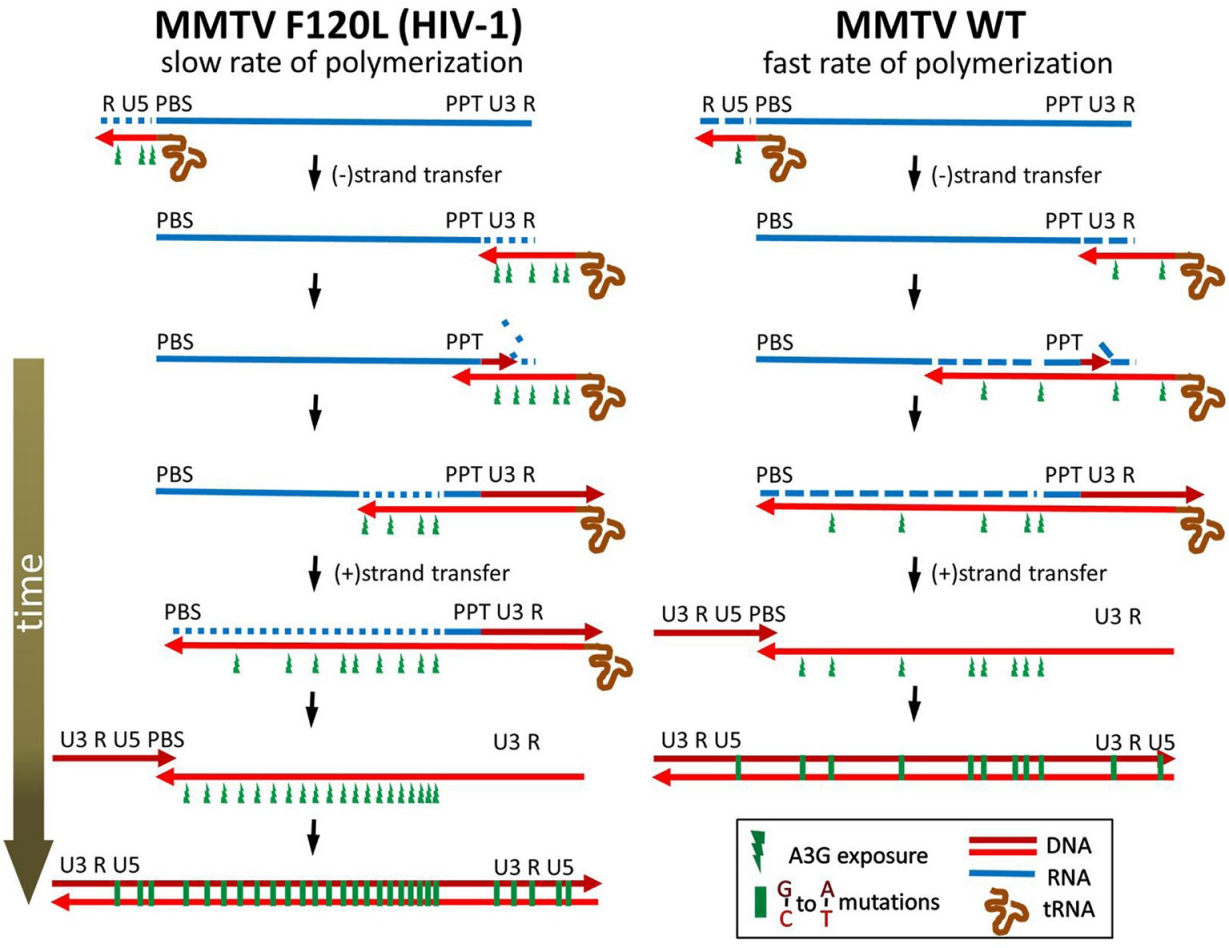
The proposed mechanism explaining lower sensitivity of MMTV to inhibition by A3s. Reverse transcriptase (RT) produces the newly synthesized minus DNA strand (light red line) using viral RNA (blue line) as a template. The RNase H domain degrades the genomic RNA template behind the polymerizing DNA polymerase domain and exposes the minus DNA strand to A3s. Viruses containing RT exhibiting a fast rate of DNA polymerization (such as MMTV) narrow a window of opportunity for A3s to deaminate the single-stranded substrate as they complete synthesis of the double-stranded DNA in a shorter time compared to viruses carrying RT with a slow rate of polymerization (e.g. MMTV F120L or HIV-1). In addition, a reduction of the polymerase activity likely changes the balance between the polymerase and RNase H activity in favor of the later leading to the generation of more primary RNA cleavages per nucleotide addition and resulting in shorter RNA/DNA duplexes behind polymerizing RT. The viral RNA is blue; the dashed blue lines indicate RNase H cleavage; minus- and plus-strand DNA are light red and dark red, respectively. Single-stranded minus-sense DNA regions exposed to APOBEC3 are shown as green flashes.

Previous reports showed that MMTV can replicate in cells producing mA3 or A3G and that proviruses recovered from these cells do not carry an extensive deamination signature of the innate immunity factors [15–17]. The mechanism underlying the ability of MMTV to escape detrimental G-to-A mutagenesis remained unknown. To shed more light on the interplay between MMTV and A3 proteins (A3s) we initially compared a Vif-deficient HIV-1 with the betaretrovirus with respect to their sensitivity to inhibition by various A3s. We found that MMTV is less sensitive to inhibition by mA3 and A3G than the lentivirus lacking Vif. The observation that MMTV partially evades A3s derived from several mammalian species suggested that the virus uses a broadly acting mechanism to escape the lethal effect of A3s. Furthermore, sensitive high-throughput sequencing confirmed the previously reported low level of G-to-A editing of the MMTV reverse transcripts produced from virions derived from cells expressing mA3 or A3G. Specifically, side-by-side comparison between MMTV and HIV-1ΔVif revealed a four-fold lower magnitude of deamination for the RT intermediates of the betaretrovirus. As both viruses carry an identical RRE-*egfp*-WPRE RNA segment, it is unlikely that the differences result from a different composition of cellular RNA binding proteins deposited on the viral RNA, a variation in RNA structure and/or thermodynamic stability that may affect RT pausing during cDNA synthesis. Furthermore, the data presented here do not support the concept that an inefficient encapsidation of A3 proteins or the expression of a gene product that counteracts the antiviral activity of the innate immunity factors is responsible for the low editing levels. Instead, the observations that a cell culture condition (e.g. a limiting dNTP concentration or the presence of a RT inhibitor) or a mutation in the RT coding region that slows down RT polymerization but does not affect A3G encapsidation, significantly increases G-to-A mutation levels and sensitizes MMTV to A3G support the view that MMTV has evolved RT to catalyze DNA polymerization with a rate that does not allow a high level of A3-mediated deamination of reverse transcription (RTN) intermediates. Given that A3s can deaminate only single-stranded (ss) DNA, the amount of time for which DNA remains single-stranded determines the period of time the ssDNA is exposed to A3s and, in turn, the sensitivity to editing by A3s. The key features of RTN which affect sensitivity to deamination are diagrammed in Fig 6 and summarized as follows. After minus strand strong-stop DNA strand transfer to the 3’ end of the viral genomic RNA, elongation of the minus DNA strand continues on a polypurine tract region (PPT) towards a primer binding site (PBS). Concomitant with the synthesis of the minus DNA strand, the RNase H activity of RT cleaves the newly copied RNA genome from the RNA/DNA heteroduplexes to short oligonucleotides, many of which may dissociate from the nascent DNA and expose it to A3s. Later, when the synthesis of the minus DNA strand reaches the PBS region at the 5‘end of viral RNA, a second strand transfer occurs. This allows the continuation of synthesis of the minus and plus DNA strands resulting in the generation of the double-stranded DNA, which is protected from A3s. The time span between degradation of template RNA and production of plus DNA strand determines the time for which a region remains single-stranded and hence vulnerable to deamination.

We propose that the fast rate of DNA polymerization detected for the WT MMTV RT shortens the time for which viral DNA remains single-stranded and hence available for A3-mediated deamination (Fig 6). This model is reminiscent of previously reported correlation between the frequency of template switching and the rate of DNA polymerization during RTN [40, 41]. Similar to our results, a cell culture condition or mutation that slows down the rate of RTN increased the frequency of template switching. The template switching events are necessary for the completion of RTN, therefore retroviral reverse transcriptases have evolved to possess low template affinity and processivity [46]. Most of the recombination events occur during minus DNA strand synthesis and rely on base pairing between the acceptor RNA sequences downstream of recombination crossover and the nascent complementary minus DNA strand sequences unmasked by RNase H activity (dynamic copy-choice model, [41]). Thus, the RNase H function of RT is essential for the production of ssDNA required for the template switching. Two different modes of RNase H activity are involved in the degradation of RNA and the generation of ssDNA. The first mode is carried out by the same RT molecule performing DNA synthesis. This mode is not sufficient to completely remove RNA from the RNA/DNA duplexes, because the polymerization rate of RTs is faster than the RNase H rate [47, 48]. The residual RNA fragments are cleaved by a polymerase-independent mode of hydrolysis catalyzed by free RTs, which are also present in reverse transcription complexes [49–52]. Conditions that reduce the rate of polymerization permit more efficient degradation of the template RNA and provide more time for hydrogen bonding between (-)ssDNA and the acceptor template, resulting in a greater frequency of template switching [40, 41].

Analogously, a mutation in the DNA polymerase domain or a cell culture condition that prolongs the elongation of the minus DNA strand increases the time during which the RNase H can degrade the RNA template and expose the nascent (-)ssDNA to A3s. In fact, both the polymerase-dependent and polymerase-independent RNase H activities may be important for the sensitivity to inhibition by A3s. Relative contribution of the two RNase H modes is not known. Previous studies showed that the magnitude of the primary RNA cleavage by RNase H during DNA synthesis differs among reverse transcriptases. For instance, the avian myeloblastosis virus reverse transcriptase cleaves RNA strand only once for every 100–200 nt, whereas the HIV-1 reverse transcriptase generates RNA fragments that are 100–120 nt long [47, 48]. Therefore, it may be possible that viruses containing reverse transcriptase with more active DNA polymerase domain relative to the RNase H domain are inhibited by A3 proteins to a lesser extent than viruses with a slower DNA polymerase domain because they generate fewer primary RNA cuts per dNTP addition. The lower frequency of RNA degradation by reverse transcriptase, which is at the same time polymerizing (polymerase-dependent RNase H activity), creates fewer primary cuts that are accessed by A3 proteins as soon as short DNA single strands are exposed. The immediate association of A3 proteins with target sites may be facilitated by nucleocapsid proteins that interact with A3 proteins and function as chaperones during reverse transcription [53]. It may also be conceivable that the primary RNA cuts do not create single-stranded DNA regions long enough to be targeted by A3 proteins. In such a scenario, the decreased frequency of deamination results from a delayed formation of sufficiently large single-stranded DNA regions by RNase H, which is not coupled to DNA synthesis (polymerase-independent RNase H activity). Virions contain an excess of reverse transcriptase molecules (50–100) that do not participate in DNA synthesis [54]. They are believed to degrade the RNA segments that remain bound to minus sense DNA following DNA synthesis accompanied with the primary RNA degradation by polymerase-dependent RNase H activity [55]. The two distinct modes of this polymerase-independent RNase H activity are referred to as RNA 5’ end-directed cleavage and internal cleavage. Internal cleavage does not involve positioning by either a DNA 3’ end or an RNA 5’ end and the most important determinant appears to be the RNA sequence in the vicinity of the cleavage site [56]. For RNA 5’ end-directed cleavage, which is kinetically favored over internal cleavage, the polymerase active site of reverse transcriptase is positioned on the DNA strand near the 5’ RNA end such that cleavages occur 13–19 nt from the RNA 5’ end [48, 57, 58]. It is conceivable that a lower frequency of the primary RNA cuts mediated by the polymerase-dependent RNase H provides fewer 5’ RNA ends accessible for reverse transcriptase. This leads to less efficient degradation of RNA by polymerase-independent RNase H. Thus, decreased polymerase-dependent RNase H activity leads to less efficient degradation of viral RNA and in turn to a reduction of the length of single-stranded DNA segments. The shorter single-stranded DNA regions narrow the window of opportunity for A3 proteins to bind and deaminate their substrate.

Although it has not been explicitly shown here, the F120L mutation in DNA polymerase domain likely decreased the ratio of DNA polymerase to RNase H activity, resulting in an increase of the number of primary RNA cleavages catalyzed by the polymerase-dependent RNase H activity [41, 48, 59, 60]. Additionally, a slower kinetics of the minus DNA strand synthesis increases the amount of time during which the polymerase-independent RNase H can further degrade the template RNA.

Our results and the proposed model are strongly supported by a recent study of the Harris group, which demonstrated that the Vif-mediated proteasomal degradation of A3G may not be the only mechanism by which HIV-1 counteracts the innate immunity factor [61]. Strikingly, they detected three Vif-null HIV-1 variants capable of replication in T cells expressing restrictive amounts of A3G with kinetics that were not markedly different from the kinetics of Vif-proficient parental HIV-1 virus. Importantly, all three variants carried mutations that facilitated packaging of Gag-Pol polyproteins resulting in elevated amounts of RT molecules in virions. The higher RT levels lead to a faster accumulation of viral DNA in infected cells, decreased opportunities for A3G-catalyzed deamination and to lover levels of G-to-A mutations and restrictions [61]. Interestingly, the study also revealed that only relatively small changes in RT activity (~3-fold increase of accumulation of the late RT products) resulting in ~2-4-fold decrease in the G-to-A levels may have large phenotypic consequences such as enabling virus replication in the presence of A3G.

Taken together, our results support the concept that the MMTV RT has evolved to protect the viral genome from excessive A3-mediated deamination and at the same time to govern template switching with an efficiency that is sufficient for the production of viral DNA at the level ensuring MMTV replication in the host.

Our findings combined with a recent study from the Harris lab points towards an emerging and potentially general mode of evasion from deleterious A3-driven hypermutation that may be employed by other RT-containing viruses and mobile elements. This may apply especially, but not exclusively, to viruses that do not encode a Vif- or Bet-like gene product or that do not escape restriction by evolving an A3-unreactive nucleocapsid. The mechanism described here suggests that some retroviruses might have evolved to coexist and perhaps even benefit from the “tolerated” sublethal mutagenic activity of A3s to fuel viral heterogeneity and immune escape.

## Materials and methods

### Plasmids

Plasmids encoding HA-tagged hA3G, hA3F, rhA3G and mA3ΔE5(Balb/c) in a pcDNA3-based vector were kindly provided by B. Cullen [62]. The HA-tagged version of mA3ΔE5(CL57BL/6) was generated by exchanging A3 coding region in the mA3ΔE5(Balb/c) plasmid [63]. Non-HA tagged versions of the A3-encoding plasmids were generously donated by M. Malim. The MMTV-based expression plasmids pRRpCeGFPWPRE25 and pCMgpRRE17 have been described previously [24]. The HIV-1 packaging (pMDLg/pRRE) and vector constructs (pRRL-cPPT-GFP), as well as the VSV-G (pHCMV-G) and Rev (pRSV-Rev) expression plasmids, have been described previously [64, 65]. To generate the MMTV packaging construct mutants PCR-based molecular cloning approaches were used (primers are shown in S7 Fig).

### Cell culture, virus production and quantification of GFP-positive cells

293T, HeLa and NMuMG cells (all obtained from ATCC) were cultured in Dulbecco’s modified Eagle’s medium containing 10% fetal calf serum (FCS). IM9 cells (obtained from ATCC) were cultured in RPMI-1640 medium with 10% FCS. For virus production, a total of 2 x 10^6^ 293T cells (each well of a six-well plate) were transfected using polyethylenimine (lin. PEI; 25 kDa) and 10 ng, 40 ng or 160 ng of an A3 expression plasmid. In addition, cells were transfected with pCMgpRRE17 (0.8 μg) and pRRpCeGFPWPRE25 (1 μg) (for MMTV production) or with pMDLg/pRRE (0.2 μg) and pRRL-cPPT-GFP (1 μg) (for HIV-1 production) [24]. Initially, we observed that the production of HIV-1 virions is more efficient compared to MMTV. Therefore, we titrated the amount of the HIV-1 packaging construct (pMDLg/pRRE) to obtain an equivalent number of viral particles in the cell culture supernatants of cells producing both viruses. Lowering of the pMDLg/pRRE amount to 0.2 μg resulted in equivalent amounts of MMTV and HIV-1 virions in cell culture media as determined by a *gfp*-specific RT-qPCR and by EM (see below; S1 and S3 Figs). All viruses were pseudotyped by addition of 0.3 μg of VSV-G expression plasmid (pHCMV-G) to transfection mixture. An efficient virus production was supported by adding Rev-encoding plasmid pRSV-Rev (0.6 μg) to the transfection cocktail. pcDNA3 filler plasmid was used to equalize DNA content. Sixteen hours post-transfection DNase I (10 U / ml) and MgCl_2_ (4 mM) were added to the cell culture medium. After one-hour incubation at 37 wC, the nuclease-containing medium was replaced with a fresh DMEM supplemented with 10% FCS. Forty-two hours post transfection the virus-containing was medium filtered (0.45 μm), serially diluted and used to infect naïve cells seeded at a density of 3 x 10^5^ cells per well in a six well plate. Polybrene (8 μg / ml) was used to facilitate the attachment of viruses to the cell membrane. The GFP-positive cells were quantified by flow cytometry forty-eight hours post infection. The virus dilution infecting up to 20% of target cells was used to calculate the virus titer and infectivity.

### Reverse transcriptase assays

To determine processivity of RTs, virions were harvested from transfected cells, concentrated by ultracentrifugation and viral loads determined by RT-qPCR and verified by EM as described above. Equivalent amounts of viral particles (adjusted to a total volume of 5 μl) were mixed with 2 x concentrated lysis buffer (40 mM Tris-HCl (pH 8), 50 mM KCl, 20 mM DTT, 0,2% Triton-X100) and incubated for 10 min at room temperature to release RTs from virions. Next, the liberated RTs were diluted (10 x) in a dilution buffer (20 mM Tris-HCl (pH 7,5), 50 mM KCL, 0,025% Triton X-100, 0,2 mM DTT, 10% glycerol) and added to a pre-formed MS2_1684R primer/MS2 bacteriophage RNA complex (in a molar excess, 250 pmol). The complex was formed by mixing the equimolar amount of the MS2 RNA (Roche) and 20-mer oligonucleotide (MS2_1684R: AGA GAA AGA TCG CGA GGA AG), heating the mixture at 65°C for 5 min and cooling it down on ice for 1 min. The final reaction mixtures contained 50 mM Tris-Cl, 75 mM KCL, 3 mM MgCl_2_, 10 mM DTT. Reaction was initiated by adding four dNTPs at a final concentration of 200 μM followed by incubation at 37°C. A DNA trap (100 pmol; S7 Fig) was added to the mixture one minute after the initiation of reaction when an RT/MS2 primer/MS2 RNA complex was formed. The reaction was carried out for an additional 15 min and terminated by heating to 95°C for 5 min. To determine the size of RT products, cDNA was purified using Monarch PCR clean up kit (NEB) and the 3’ends were A-tailed using a terminal transferase (Roche). The reaction mixture contained the MS2 cDNA, 1 x terminal transferase buffer (NEB), CoCl_2_ (250 μM) and dATP (100 μM). Samples were primed at 94°C for 3 min followed by 1 min on ice prior to addition of the terminal transferase (1 U) and incubation at 37°C for 30 min. Following inactivation of the terminal transferase (70°C for 10 min), 1/10 of the A-tailing reaction volume was used for a PCR with oligo(dT)-anchor- (GAC CAC GCG TAT CGA TGT CGA C-(T)_30_-V) and MS2-specific (1583R: CGG CTA CAG GAA GCT CTA C) primers. PCR products were visualized on 1.5% agarose gels.

The rate of DNA polymerization during RTN was determined using virion-derived reverse transcriptases and the MS2 RNA as template. Concentrated viral stocks were prepared, quantified and the amount used for testing was normalized as described above. Lysed and diluted virus preparations were added to pre-annealed MS2_1934R primer/MS2 RNA (25pmol) (MS2_1934R: GGA TCC CAT GAC AAG GAT TT). RTN that was carried out in the absence of the trap in a reaction mixture containing 50 mM Tris-Cl, 75 mM KCL, 3 mM MgCl_2_, 10 mM DTT and 10 μM dNTPs was terminated 0 min, 2.5 min, 5 min, 10 min or 40 min after the RTN initiation (95°C, 5 min). Next, a PCR was used to detect the presence of 1.4 kb- and 0.4 kb-long cDNA products with primer pairs MS2_1252R (TTC CGC CAT TGT CGA CGA G) plus MS2_512F (TCC GCT ACC TTG CCC TAA AC) and MS2_1934R plus 1523F (TTA AGG CAA TGC AAG GTC TC), respectively.

### Quantification of late RT products

Unconcentrated virus stocks were used for infection of target 293T cells seeded in 6 well plates. Virus inoculum was removed two hours post-infection, cells rinsed with PBS and fresh DMEM supplemented with 10% FCS was added. Total cellular DNA was harvested from the infected cells at desired time points using DNeasy Blood and Tissue kit (Qiagen) and used in a qPCR with MMTV-specific primers designed to amplify the late RT products (1176F: TTC CTG ACT TGG TTT GGT ATC AAA TG and 1495R: AGA CAA GGG TCA CTT ATC CGA G) using a GoTaq Probe qPCR master mix (Promega). A 321 bp-long PCR product can be detected only after the second DNA strand transfer because the viral RNA is produced from a hybrid LTR consisting of the U3 region derived from RSV and RU5 derived from MMTV. Amplification results were normalized to *actin* DNA levels determined in the samples with actin5F (CTT CTG CCG TTT TCC GTA GG) and actin3R (TGG GAT GGG GAG TCT GTT CA) primers.

### Construction of RT mutants

Oligonucleotides designed to introduce individual mutations (S7 Fig) to the RT-encoding portion of the *pol* gene in the MMTV packaging construct, pCMgpRRE17, were used for PCR (Q5 High-fidelity DNA polymerase, NEB). The products generated using F119_R and one of the F119X_F (X = V, L, W, P or Y) or F120Z_F (Z = V, L, W, P, Y or A) primers were digested with Bgl II and ligated with a large fragment generated from the packaging construct by Bgl II digestion. Mutations Q155N and K70R were first introduced to a long PCR product (2.6 kb) by an overlap extension PCR. Next, the amplicon was digested with XhoI and EcoRI and ligated with a large fragment resulting from XhoI-EcoRI digestion of the packaging construct. The presence of desired mutations was verified by DNA sequencing.

### The packaging of A3 proteins into virions

The packaging of A3 proteins into MMTV and HIV-1 virions was analyzed as follows. 293T cells were transfected as described above. Virus-containing supernatant was collected, clarified by low-speed centrifugation, filtered through a 0.45 μm filter and layered onto a 20% sucrose cushion. Virions were pelleted by ultracentrifugation (50 000 g, 2h, 4°C), re-suspended in PBS (50 μl) and stored at -80°C. An aliquot was used for RNA extraction as described above. DNase-treated (Turbo DNA-free kit; Ambion) viral RNA was quantified using the TaqMan RT-PCR as described above. The determined virus loads were used to normalize inputs for an immunoblot analysis.

### Immunoblot analyses

Aliquots containing equal amounts of total proteins from cell lysates (10 μg of protein, DC protein assay, BioRad) and virion lysates (*egfp* levels normalized) were subjected to gel electrophoresis. After blotting to a PVDF membrane, proteins were detected using an antibody specific to HA-tag (Roche), hA3G (Abcam), HIV-1 p24 (Polymun), MMTV CA(p27) (a gift from A. Mason), actin (Sigma-Aldrich), HSP90 (Santa Cruz). Antibody-reactive proteins were detected with horseradish peroxidase-conjugated secondary antibody (DAKO) and ECL plus substrate (GE Healthcare).

### Trans-complementation assay

HIV-1 vector was produced by transient transfection as described above. Transfection cocktail contained an A3G-expression plasmid (40 ng) or parental pcDNA3 (40 ng; no A3G control). In addition, the transfection mixture was in respective cases supplemented with the complete molecular clone of MMTV pGR102 [34] (0.2 μg; a gift from B. Salmons) or with pcDNA3 (0.2 μg). As a positive control Vif-expression plasmid (0.2 μg; a gift from M. Malim) was used. The infectivity of viral vectors was assessed on naïve 293T cells.

### Sequence analysis of proviral DNA

The genomic DNA was extracted from cells (2dpi) infected with MMTV WT, MMTV F120L or HIV-1 produced in the absence or presence of hA3G or mA3 (40 ng). Segments of proviral DNA common for all viruses were amplified using the Q5 High-Fidelity DNA polymerase (NEB) and the primers: WPRE_OUT_EcoRI_F1 (5'-GCC CCG GAA TTC TGC CCG ACA ACC ACT ACC T-3') or and WPRE_OUT_XhoI_R1 (5'-AAG GGA CTC GAG ACT CGT CTG AGG GCG AAG-3'). The amplicons were digested with XhoI and EcoRI, cloned into pcDNA3 linearized with the same enzymes, transformed into TOP 10 cells (Invitrogen) and plated onto twenty agar plates. Plasmid DNA was extracted from colonies scraped from the plates. The resulting library was sequenced by GS FLX system using pcDNA3-specific primers (S7 Fig). High-quality reads were aligned to vector sequence using DNASTAR mapper, exported to Microsoft Excel and manually curated. The frequency of G-to-A transitions was calculated using VBA scripts. The frequency of G-to-A transitions in the sequenced segments of the MMTV provirus was compared to the frequency of the G-to-A mutations in the HIV-1 genomes and the P values of any differences were determined using the χ^2^ test. Sequence analysis of the F120L mutant vs WT MMTV proviruses was performed using amplicons generated with WPRE-specific primers shown in S7 Fig. The amplicons were subjected to high throughput sequencing (MiSeq, 300 bp). The sequences were demultiplexed mapped to the reference genome using a Bowtie2 algorithm and 2500 randomly selected sequences were used for a Hypermut analysis. Raw MiSeq sequencing files are publicly available at the European Nucleotide Archive (ENA; www.ebi.ac.uk/ena) under accession numbers: ERS2953115—ERS2953118.

### Direct repeat deletion assay

The direct repeat deletion assay was performed as described previously [41]. Briefly, an MMTV-based vector (pRRpCeWPRE25_GFFP) containing an *egfp* gene carrying a 269 bp-long duplication of an internal segment and a 25 bp-long spacer between the repeats (*egffp*) was constructed using a PCR-based approach with the following primer pairs: GFP_F1_BamHI (5’-CGA CTC TAG AGG ATC CAC CG-3’) and GFP_R1_Xho (5’-GAA TTC CTC GAG TTG AAG TCG ATG CCC TTC AG-3’); GFP_F2_link_Xho (5’-GAA TTC CTC GAG GAT ATC TAA GTG ACT CCT GAC CCT GAA GTT CAT CTG-3’) and GFP_R2_BsrGI (5’-CCG CTT TAC TTG TAC AGC TC-3’). Amplicons were ligated and introduced into BamH I-BsrG I-digested pRRpCeGFPWPRE25 vector. Next, an IRES-puro cassette was amplified and introduced into BsrG I-Not I-cleaved pRRpCeWPRE25_GFFP. The resulting vector plasmid carrying *egffp* and *pac* (puromycin resistance) genes was named pRRpCgW_GFFPiresPURO. Virus-containing *egffp*-IRES-puro cassette was produced from 293T cells transfected with the vector plasmid, helper plasmids pCMgpRRE17 and pRSV-Rev and finally with plasmid encoding VSV-G Env. Virus-containing supernatant was filtered, diluted to achieve the multiplicity of infection of < 0.01 and then used for infection of 293T target cells. Cells were selected for resistance to puromycin (2 μg/ml) and the resulting colonies were pooled and analyzed by flow cytometry.

### Quantification of retrovirus particles by RT-qPCR and transmission electron microscopy (TEM)

To quantify viral particles produced from transfected cells, the cell culture supernatants were treated with DNase I (10 U /ml, Roche) in the presence of MgCl_2_ (4 mM) for 1 h at 37°C prior to the extraction of viral RNA using QIAamp viral RNA kit (Qiagen). For some applications (immunoblots, *in vitro* reverse transcriptase assays), the virus preparations were first concentrated by ultracentrifugation (see below) prior to RNA extraction. The extracted RNA was treated with TURBO DNA-free (Ambion) then incubated with MuLV RT at 50°C for 45 min (RT+ samples). Parallel samples were incubated in the absence of MuLV RT to determine carryover contamination from plasmid DNA (RT- samples). The cDNA was used in a qPCR with *egfp*-specific primers (214F:GCA GTG CTT CAG CCG CTA C; 309R:AAG AAG ATG GTG CGC TCC TG), a TaqMan probe (6FAM-CCG ACC ACA TGA AGC AGC ACG ACT T-TAMRA) and a Luna Universal qPCR Master mix (NEB). The cycling conditions were: initial denaturation at 95°C for 1min followed by 40 cycles of denaturation at 95°C for 15 s and annealing/extension at 60°C for 30 s. For quantification by TEM, all wells of a six-well plate were transfected as described above. Medium was collected 42 h after transfection (12 ml per virus), clarified by a low speed centrifugation (1000 g, 10 min) and by filtration (0.45 μm). Viral particles were pelleted by ultracentrifugation at 50 000 g for 2h (SW41; 4°C) and re-suspended in 1/200 of the initial volume (PBS). Virions were deposited on Formvar carbon-coated copper grids and stained with 4% phosphotungstic acid (10 min at room temperature). Fifty grid spaces were inspected by TEM (TE 900, Carl Zeiss), any retrovirus-like particles were counted and a mean number of virions per slide was calculated.

### Treatment of target cells with hydroxyurea and determination of intracellular dATP content

Treatment with hydroxyurea (HU) was performed as described previously (Julias et al., 1998). Briefly, the target cells were incubated in the HU-lacking or HU-containing medium (0.2 mM HU) 4 h before infection as well as 20 h after infection to ensure that reverse transcription is ongoing in the cells with altered dNTP pools. To verify that the treatment inhibited ribonucleotide reductase leading to a decrease of dNTP concentrations, the intracellular dATP levels were quantified as described previously (Wilson et al., 2011). Briefly, 0.6 x 10^6^ cells cultured in the HU-lacking or HU-containing medium were trypsinized, washed twice in PBS and pelleted by a low-speed centrifugation. The pellets were re-suspended in cold 60% methanol, incubated at 95°C for 3 min and sonicated for 30 s. The extracts were centrifuged (16 000g, 5min, 4°C) and the supernatant passed through Amicon Ultra 0.5 ml (MWCO 3 kDa). The filtrate was evaporated under centrifugal vacuum, the pellet re-suspended in nuclease-free water and used for a primer extension reaction in an AB 7500 Real-time PCR System with the NDP-1 primer (CCG CCT CCA CCG CC), FAM-dATP probe (6FAM/TGG TCC GTG/ZEN/GCT TGT GCG TGC GT/IBFQ), dATP-DT2 template (ACG CAC GCA CAA GCC ACG GAC CAA ATA AAT AAA GGC GGT GGA GGC GG), dNTPs (excluding dATP), AmpliTaq Gold polymerase and 10 x PCR buffer II. The polymerase was activated at 95°C for 10 min the primer extension was carried out at 60°C for 30 min with raw fluorescence spectra for 6-FAM acquired every 5 min. For treatment with 3’-azido-3’-deoxythymidine (AZT, 70 nM), target cells were infected with MMTV and at the same time, the drug was added to the inoculum. Fresh medium containing AZT was added 3 h post infection. Note that AZT was titrated and the concentration that was used (70 nM) reduced the titer of MMTV to approximately 50% of the control virus titers (S8 Fig).

### Quantification of retrovirus particles by RT-qPCR and transmission electron microscopy (TEM)

To quantify viral particles produced from transfected cells, the cell culture supernatants were treated with DNase I (10 U /ml, Roche) in the presence of MgCl_2_ (4 mM) for 1 h at 37°C prior to the extraction of viral RNA using QIAamp viral RNA kit (Qiagen). For some applications (immunoblots, *in vitro* reverse transcriptase assays), the virus preparations were first concentrated by ultracentrifugation (see below) prior to RNA extraction. The extracted RNA was treated with TURBO DNA-free (Ambion) then incubated with MuLV RT at 50°C for 45 min (RT+ samples). Parallel samples were incubated in the absence of MuLV RT to determine carryover contamination from plasmid DNA (RT- samples). The cDNA was used in a qPCR with *egfp*-specific primers (214F:GCA GTG CTT CAG CCG CTA C; 309R:AAG AAG ATG GTG CGC TCC TG), a TaqMan probe (6FAM-CCG ACC ACA TGA AGC AGC ACG ACT T-TAMRA) and a Luna Universal qPCR Master mix (NEB). The cycling conditions were: initial denaturation at 95°C for 1min followed by 40 cycles of denaturation at 95°C for 15 s and annealing/extension at 60°C for 30 s.

For quantification by TEM, all wells of a six-well plate were transfected as described above. The medium was collected 42 h after transfection (12 ml per virus), clarified by a low-speed centrifugation (1000 g, 10 min) and by filtration (0.45 μm). Viral particles were pelleted by ultracentrifugation at 50 000 g for 2h (SW41; 4°C) and re-suspended in 1/200 of the initial volume (PBS). Virions were deposited on Formvar carbon-coated copper grids and stained with 4% phosphotungstic acid (10 min at room temperature). Fifty grid spaces were inspected by TEM (TE 900, Carl Zeiss), any retrovirus-like particles were counted and a mean number of virions per slide was calculated.

## Supporting information

S1 Fig Monitoring of HIV-1 and MMTV virus particle productions by RT-qPCR with *egfp*-specific primers. (A) Dose titration of HIV-1 packaging construct to normalize HIV-1 virus production to the levels obtained with MMTV packaging plasmid (B) Verification of equal virus production from three independent transfections.
(PPTX)

S2 Fig Graphic representation of the G-to-A mutations (compared to ref. [vector sequences]) present in the WPRE region. The analysis was performed using the HYPERMUT 2.0 program (https://www.hiv.lanl.gov/content/sequence/HYPERMUT/background.html). All possible G-to-A changes in the context of the WPRE sequence present in both the HIV-1ΔVif and MMTV viruses are shown with the dinucleotide context color.
(PPTX)

S3 Fig Virus quantification by TaqMan RT-qPCR followed by transmission electron microscopy (TEM). (A) Representative examples of virus quantification using AB 7500 Real-Time PCR System with *egfp*-specific primers and a TaqMan probe. The primers and probe allow quantification of the genomic RNA in the MMTV WT (red and brown lines), MMTV F120L (light and dark green lines) and HIV-1 (blue and cyan lines) virus preparations (performed in duplicates). Plasmid DNA contaminations were removed by treating the cell culture supernatant with DNase I in the presence of MgCl_2_ and by removing the residual DNA in the extracted RNA by the TURBO DNA-free reagent. The treated RNA was then reverse-transcribed to cDNA (RT step included, left figure) and subjected to TaqMan PCR. Parallel RT minus samples were analyzed to determine the level of plasmid DNA carryover (right). A shift in the threshold cycle number (~10 cycles) between TURBO DNA-free treated and untreated samples indicates an efficient removal of the plasmid DNA. An equivalent amount of viral RNAs in all three virus preparations is indicated by the identical threshold cycle detected for the MMTV WT, F120L, and HIV-1 (left). (B) Representative TEM photographs used for quantification of MMTV WT, MMTV F120L, and HIV-1 virions. Viruses were harvested from transfected 293T cells, concentrated (200-fold) by ultracentrifugation and aliquots frozen at -80°C. One aliquot was used for viral RNA quantification as shown in (A). Another aliquot was used for TEM. To this end, virus re-suspended in PBS was applied onto a formvar-carbon-coated copper grid, stained with 4% phosphotungstic acid (10 min) and viewed by a transmission electron microscope at 10 000 x magnification. Fifty consecutive fields of view were photographed and virus particles counted (bar = 100 nm). Mean number of virions per slide +/- SEM is shown in (C). Differences between groups were calculated by a two tailed Student’s t-test in GraphPad Prism. Samples with statistically insignificant differences (P>0.05) were used for further analyses (n.s., not significant).
(PPTX)

S4 Fig The DNA polymerase domain mutants of MMTV RT. (A) Amino acid sequence alignment of three retroviral reverse transcriptase sequences (the DNA polymerase domains). Sequences were retrieved from UniProt and NCBI database (HIV-1: HXB2 strain, UniProtKB- P04585; MLV: UniProtKB-P03355; MMTV: BR6 strain, NCBI # M15122). The sequences were aligned using CloneManager software package and manually curated according to Barber et al., 1990 (Barber, A. M., et al. (1990). "HIV-1 reverse transcriptase: structure predictions for the polymerase domain." AIDS Res Hum Retroviruses **6**(9): 1061–1072.). The residues subjected to mutagenesis are depicted in red. The dNTP binding sites are shown in blue. The consensus sequence is shown in green. “-” represents gaps in the specified position. (B) Infectivity of the mutants relative to the wild-type RT determined in a single round infection experiment.
(PPTX)

S5 Fig Graphic representation of the G-to-A mutations present in the WPRE region. The analysis was performed using the HYPERMUT 2.0 program. 2500 (2215 seq. for mA3/F120L) randomly selected sequences were used for analysis. All possible G-to-A changes in the context of the WPRE sequence present in both the MMTV WT and MMTV F120L viruses are shown with the dinucleotide context color. The table below each graph depicts the percent of MMTV sequences carrying the indicated number of mutations within the 270 bp sequence.
(PPTX)

S6 Fig Decreased dATP levels in cells treated with a drug inhibiting ribonucleotide reductase, hydroxyurea (HU). (A) Naïve 293T cells were seeded in a six-well plate at a concentration of 3 x 10^5^ cells per well. HU was added next day four hours prior to dNTPs extraction at a final concentration of 0.2 mM. Next, the HU- and mock-treated cells were counted and 0.6 x 10^6^ cells were used for dNTPs extraction followed by dATP quantification as described in Methods. The upper panel shows a schematic diagram of the fluorescence-based assay for the quantification of dATPs. A primer (red arrow) is extended in the presence of dTTP, dCTP and dGTP and the cellular dNTP extract (containing dATP to be measured). When Taq polymerase reaches a probe (green line), its exonuclease activity releases the 6-FAM-labelled nucleotide from the probe resulting in an increase of fluorescence signal. When the dATP from cellular extract becomes exhausted primer extension is terminated leading to no further increase of the fluorescence intensity due to a quenching of fluorescence in the intact probe. The lower panel shows the result of two independent measurements as values normalized to 100% (ctrl = HU-untreated sample).
(B) The EGFP expression is delayed in cells treated with hydroxyurea (HU). Cells treated with HU (0.2 mM), when compared to mock-treated cells, show morphological changes including enlarged cell body and nucleus size (see phase contrast figures; 20 hpi). They also prolong their doubling time resulting in a reduced number of cells per well (bottom figures). The onset of EGFP expression was delayed in the HU-treated cells infected with the MMTV-eGFP virus. Whereas the EGFP expression could be detected in mock-treated cells already 24 hpi, the EGFP signal was detectable in HU-treated cells only at later time points (48 hpi).
(PPTX)

S7 Fig Oligonucleotides used for a site-directed mutagenesis, deep sequencing and as a trap for reverse transcriptase assays. (PPTX)

S8 Fig Inhibition of MMTV infectivity by AZT. AZT at the indicated concentration was added to the target cells together with virus inoculum. Virus infectivity is shown as the percentage of GFP positive cell at each drug concentration relative to the proportion of GFP cells infected without AZT. The assay was repeated three times and the error bars represent +/- SD. The calculated IC_50_ was 70 μM.
(PPTX)
