# Supplementary figures and images for: A high rate of polymerization during synthesis of mouse mammary tumor virus DNA alleviates hypermutation by APOBEC3 proteins
Source: PLoS Pathog. 2019 Feb 15;15(2):e1007533. doi: 10.1371/journal.ppat.1007533 (PMC6395001; doi:10.1371/journal.ppat.1007533)

## Slide 1
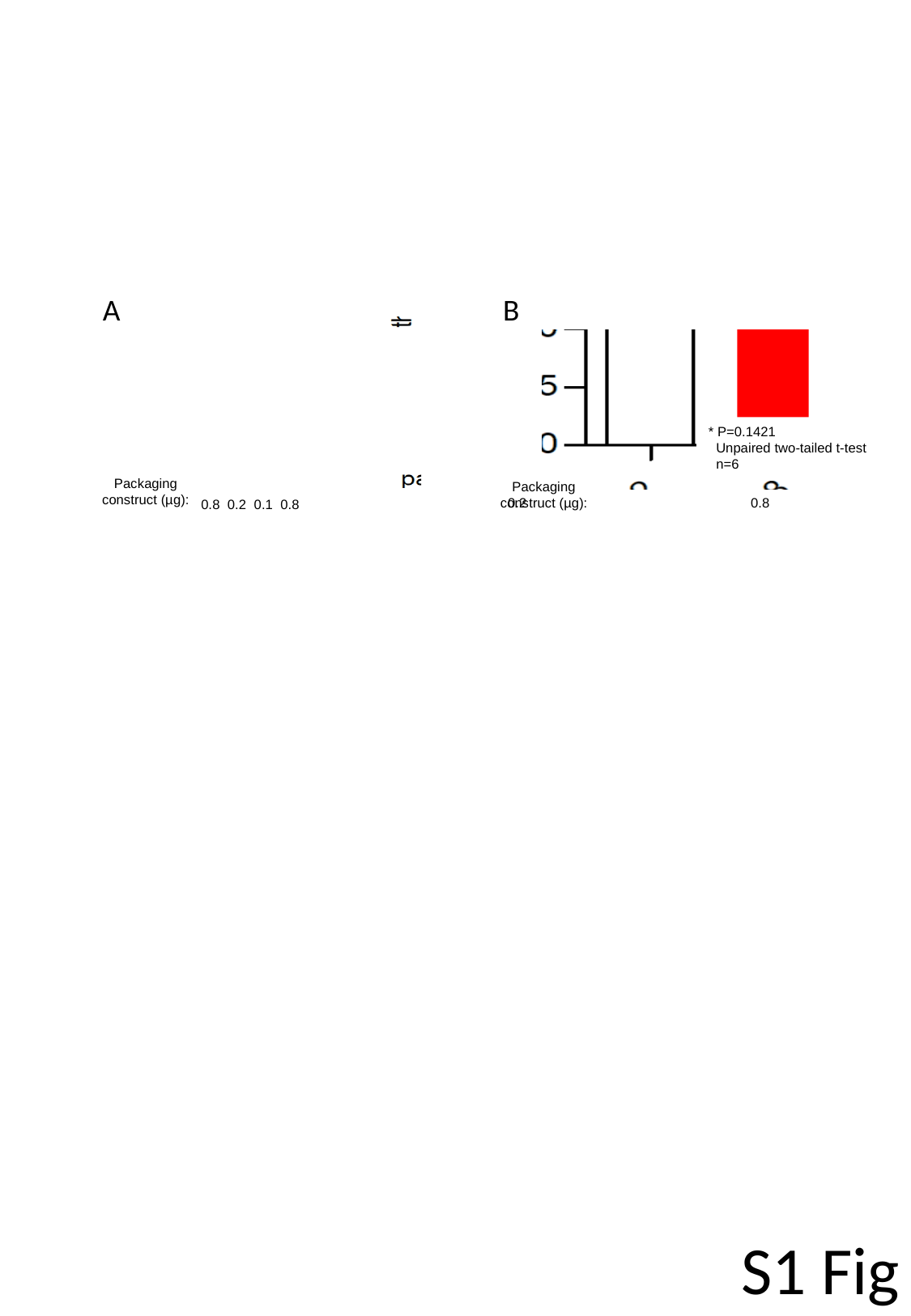

A
B
Packaging
construct (µg):
0.8 0.2 0.1 0.8
* P=0.1421
 Unpaired two-tailed t-test
 n=6
Packaging
construct (µg):
0.2		0.8
S1 Fig

Supplement: S1 Fig — (A) Dose titration of HIV-1 packaging construct to normalize HIV-1 virus production to the levels obtained with MMTV packaging plasmid (B) Verification of equal virus production from three independent transfections. (PPTX) [file ppat.1007533.s001.pptx]

## Slide 1
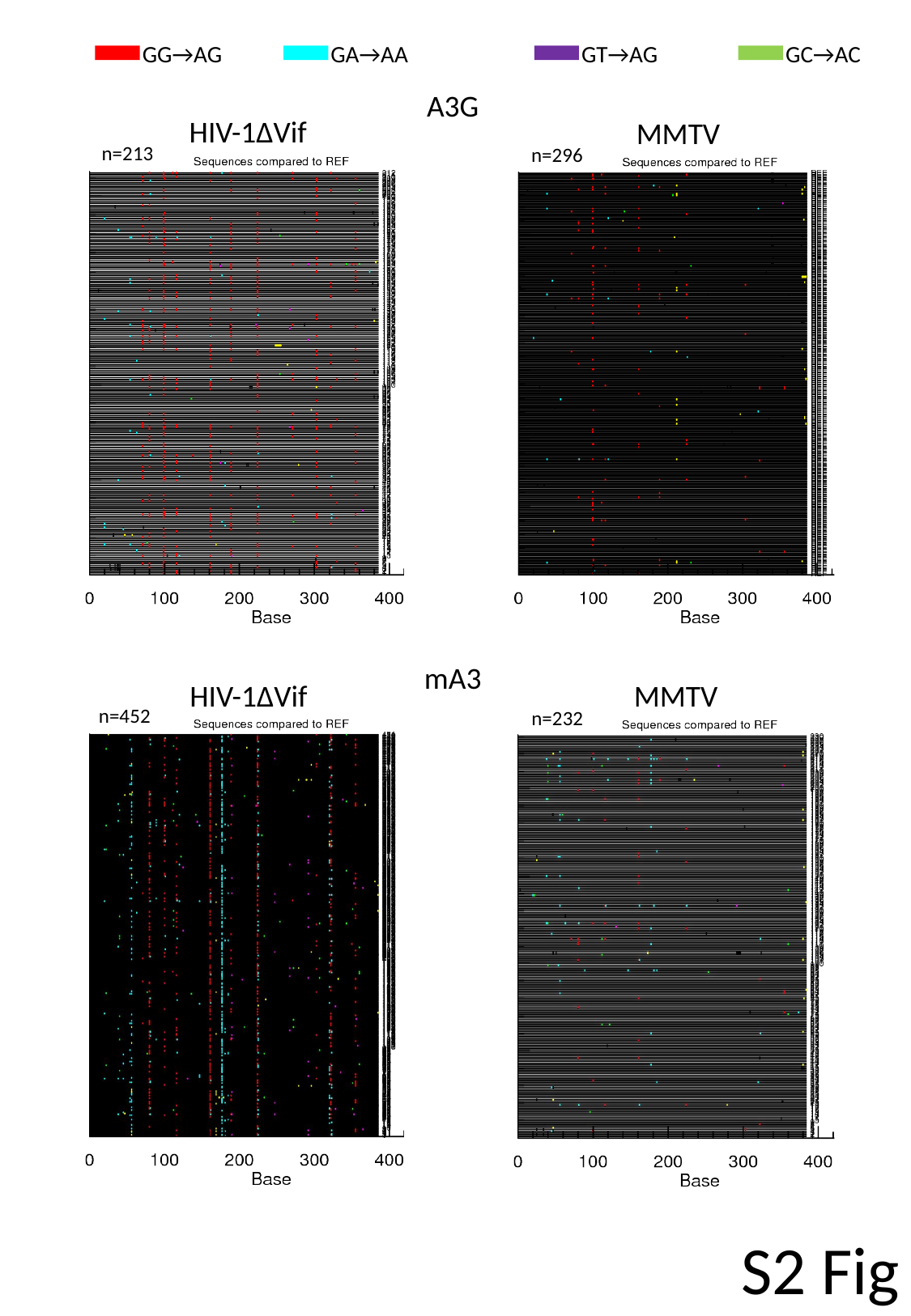

GG→AG
GA→AA
GT→AG
GC→AC
A3G
HIV-1ΔVif
n=213
MMTV
n=296
mA3
HIV-1ΔVif
n=452
MMTV
n=232
S2 Fig

Supplement: S2 Fig — The analysis was performed using the HYPERMUT 2.0 program (https://www.hiv.lanl.gov/content/sequence/HYPERMUT/background.html). All possible G-to-A changes in the context of the WPRE sequence present in both the HIV-1ΔVif and MMTV viruses are shown with the dinucleotide context color. (PPTX) [file ppat.1007533.s002.pptx]

## Slide 1
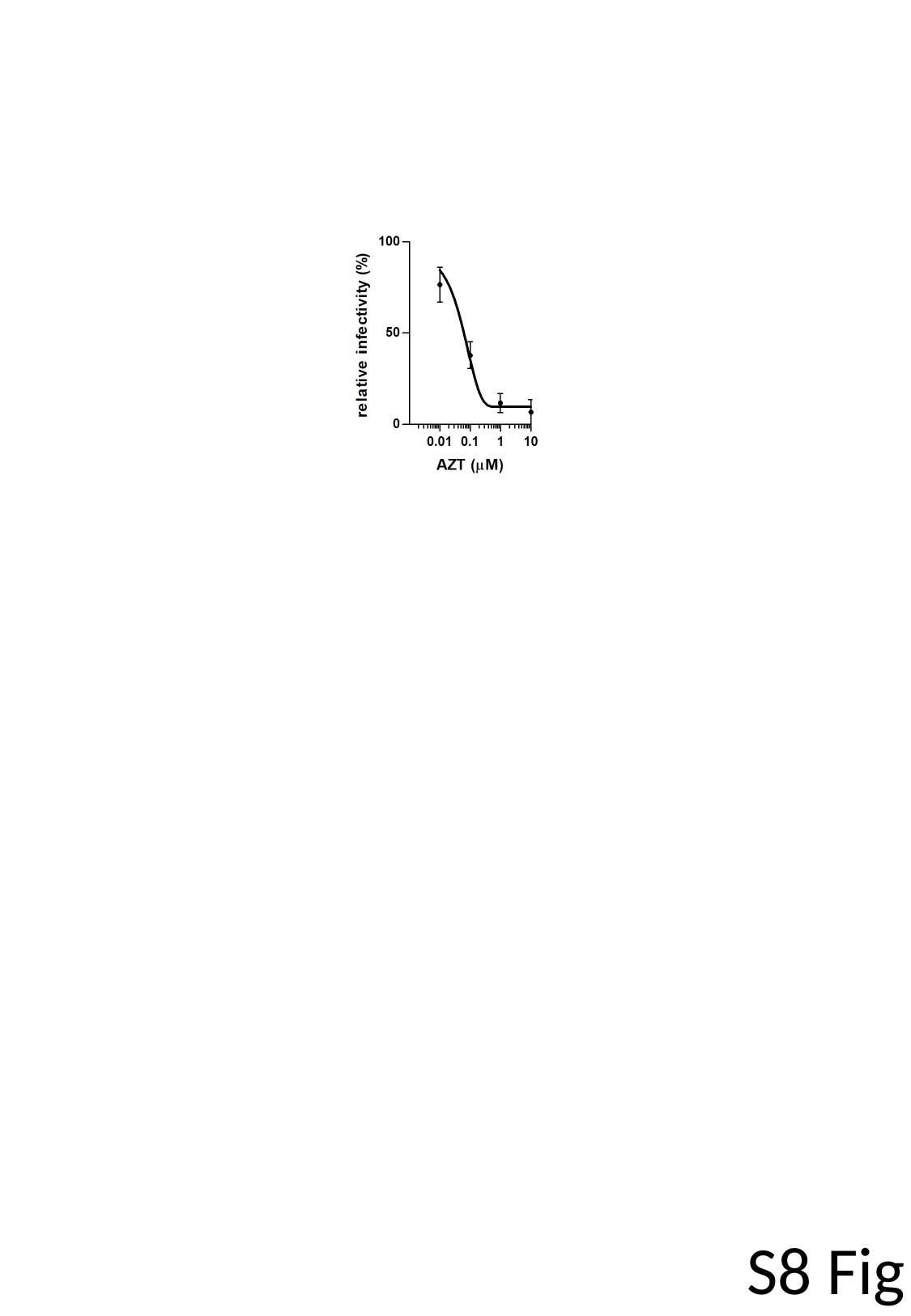

S8 Fig

Supplement: S8 Fig — AZT at the indicated concentration was added to the target cells together with virus inoculum. Virus infectivity is shown as the percentage of GFP positive cell at each drug concentration relative to the proportion of GFP cells infected without AZT. The assay was repeated three times and the error bars represent +/- SD. The calculated IC50 was 70 μM. (PPTX) [file ppat.1007533.s008.pptx]
