# Supplementary material for: A high rate of polymerization during synthesis of mouse mammary tumor virus DNA alleviates hypermutation by APOBEC3 proteins
Source: PLoS Pathog. 2019 Feb 15;15(2):e1007533. doi: 10.1371/journal.ppat.1007533 (PMC6395001; doi:10.1371/journal.ppat.1007533)
Supplement: S3 Fig — (A) Representative examples of virus quantification using AB 7500 Real-Time PCR System with egfp-specific primers and a TaqMan probe. The primers and probe allow quantification of the genomic RNA in the MMTV WT (red and brown lines), MMTV F120L (light and dark green lines) and HIV-1 (blue and cyan lines) virus preparations (performed in duplicates). Plasmid DNA contaminations were removed by treating the cell culture supernatant with DNase I in the presence of MgCl2 and by removing the residual DNA in the extracted RNA by the TURBO DNA-free reagent. The treated RNA was then reverse-transcribed to cDNA (RT step included, left figure) and subjected to TaqMan PCR. Parallel RT minus samples were analyzed to determine the level of plasmid DNA carryover (right). A shift in the threshold cycle number (~10 cycles) between TURBO DNA-free treated and untreated samples indicates an efficient removal of the plasmid DNA. An equivalent amount of viral RNAs in all three virus preparations is indicated by the identical threshold cycle detected for the MMTV WT, F120L, and HIV-1 (left). (B) Representative TEM photographs used for quantification of MMTV WT, MMTV F120L, and HIV-1 virions. Viruses were harvested from transfected 293T cells, concentrated (200-fold) by ultracentrifugation and aliquots frozen at -80°C. One aliquot was used for viral RNA quantification as shown in (A). Another aliquot was used for TEM. To this end, virus re-suspended in PBS was applied onto a formvar-carbon-coated copper grid, stained with 4% phosphotungstic acid (10 min) and viewed by a transmission electron microscope at 10 000 x magnification. Fifty consecutive fields of view were photographed and virus particles counted (bar = 100 nm). Mean number of virions per slide +/- SEM is shown in (C). Differences between groups were calculated by a two tailed Student’s t-test in GraphPad Prism. Samples with statistically insignificant differences (P>0.05) were used for further analyses (n.s., not significant). ( [file ppat.1007533.s003.pptx]

## Slide 1
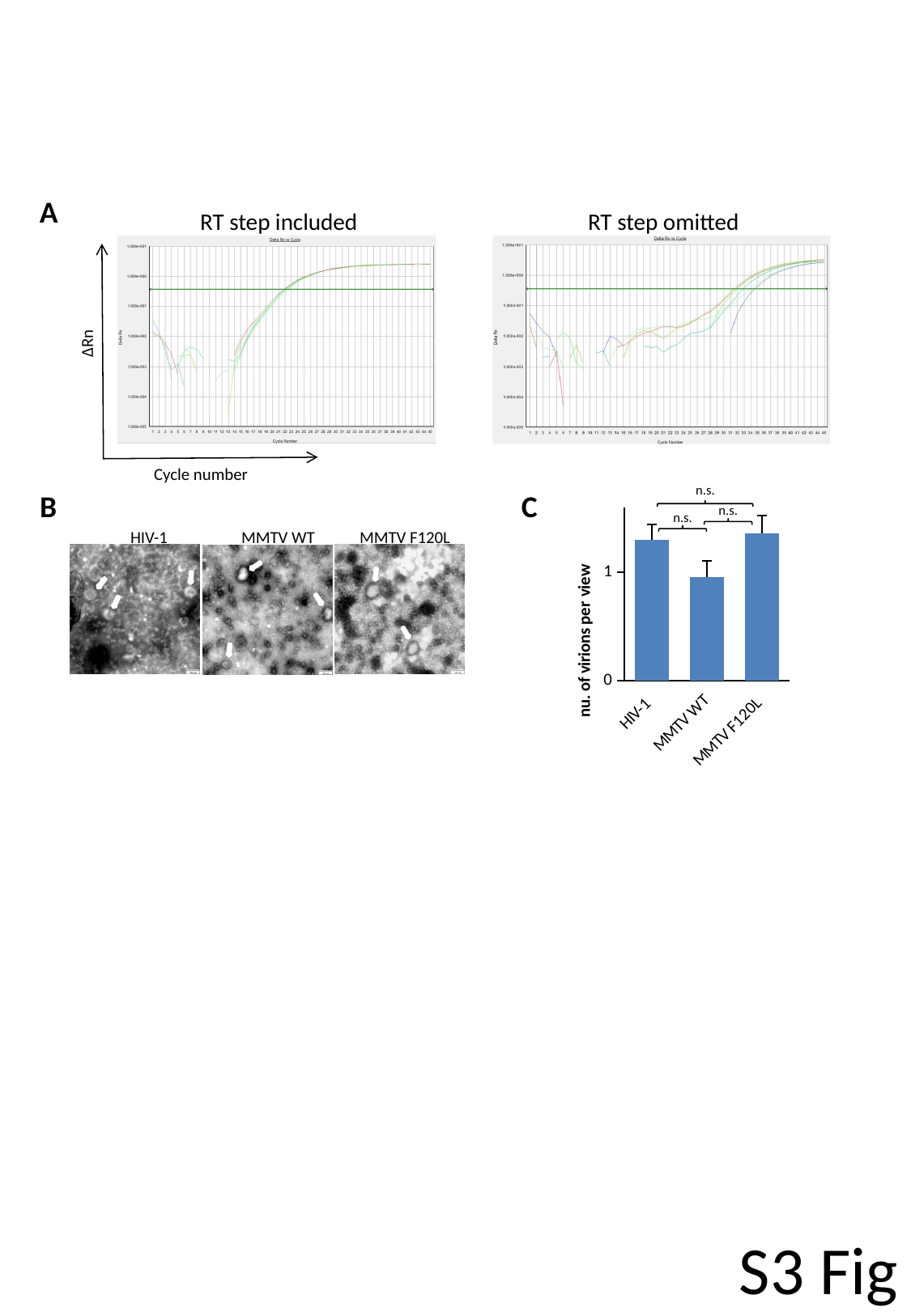

A
RT step included
RT step omitted
ΔRn
Cycle number
n.s.
n.s.
### Chart
| Category | |
|---|---|
| HIV-1 | 1.3 |
| MMTV WT | 0.96 |
| MMTV F120L | 1.36 |n.s.
B
C
MMTV WT
MMTV F120L
HIV-1
S3 Fig
