# Supplementary material for: A high rate of polymerization during synthesis of mouse mammary tumor virus DNA alleviates hypermutation by APOBEC3 proteins
Source: PLoS Pathog. 2019 Feb 15;15(2):e1007533. doi: 10.1371/journal.ppat.1007533 (PMC6395001; doi:10.1371/journal.ppat.1007533)
Supplement: S4 Fig — (A) Amino acid sequence alignment of three retroviral reverse transcriptase sequences (the DNA polymerase domains). Sequences were retrieved from UniProt and NCBI database (HIV-1: HXB2 strain, UniProtKB- P04585; MLV: UniProtKB-P03355; MMTV: BR6 strain, NCBI # M15122). The sequences were aligned using CloneManager software package and manually curated according to Barber et al., 1990 (Barber, A. M., et al. (1990). "HIV-1 reverse transcriptase: structure predictions for the polymerase domain." AIDS Res Hum Retroviruses 6(9): 1061–1072.). The residues subjected to mutagenesis are depicted in red. The dNTP binding sites are shown in blue. The consensus sequence is shown in green. “-” represents gaps in the specified position. (B) Infectivity of the mutants relative to the wild-type RT determined in a single round infection experiment. (PPTX) [file ppat.1007533.s004.pptx]

## Slide 1
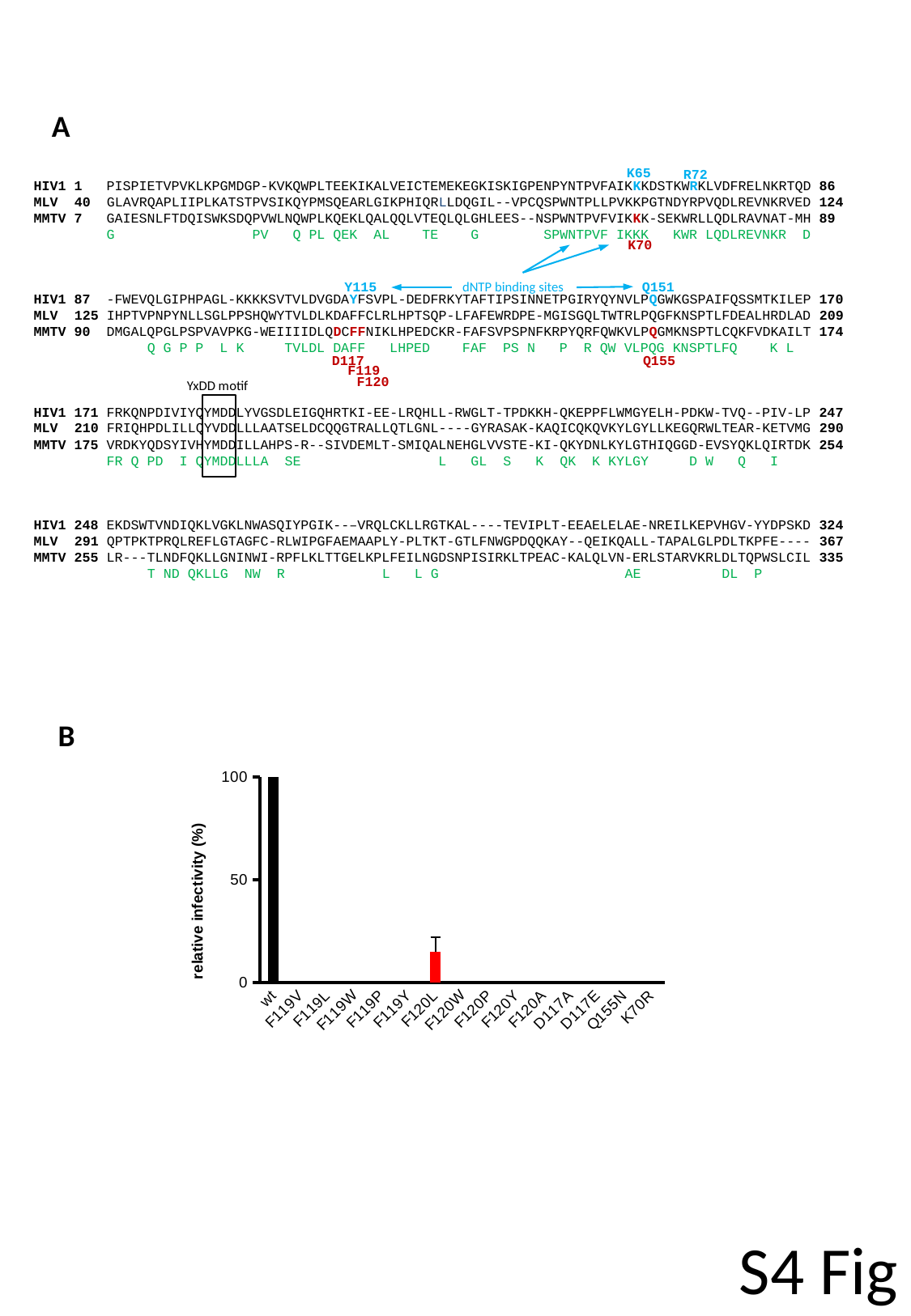

A
K65
r72
HIV1 1 pispietvpvklkpgmdgp-kvkqwplteekikalveictemekegkiskigpenpyntpvfaikkkdstkwrklvdfrelnkrtqd 86
MLV 40 GLAVRQAPLIIPLKATSTPVSIKQYPMSQEARLGIKPHIQRLldqgil--vpcqspwntpllpvkkpgtndyrpvqdlrevnkrved 124
MMTV 7 gaiesnlftdqiswksdqpvwlnqwplkqeklqalqqlvteqlqlghlees--nspwntpvfvikKk-sekwrllqdlravnat-mh 89
 G PV Q PL QEK AL TE G SPWNTPVF IKKK KWR LQDLREVNKR D
HIV1 87 -fwevqlgiphpagl-kkkksvtvldvgdayfsvpl-dedfrkytaftipsinnetpgiryqynvlpqgwkgspaifqssmtkilep 170
MLV 125 ihptvpnpynllsglppshqwytvldlkdaffclrlhptsqp-lfafewrdpe-mgisgqltwtrlpqgfknsptlfdealhrdlad 209
MMTV 90 dmgalqpglpspvavpkg-weiiiidlqdcffniklhpedckr-fafsvpspnfkrpyqrfqwkvlpqgmknsptlcqkfvdkailt 174
 Q G P P L K TVLDL DAFF LHPED FAF PS N P R QW VLPQG KNSPTLFQ K L
HIV1 171 frkqnpdiviyqymddlyvgsdleigqhrtki-ee-lrqhll-rwglt-tpdkkh-qkeppflwmgyelh-pdkw-tvq--piv-lp 247
MLV 210 friqhpdlillqyvddlllaatseldcqqgtrallqtlgnl----gyrasak-kaqicqkqvkylgyllkegqrwltear-ketvmg 290
MMTV 175 vrdkyqdsyivhymddillahps-r--sivdemlt-smiqalnehglvvste-ki-qkydnlkylgthiqgGd-evsyqkLqirtdk 254
 FR Q PD I QYMDDLLLA SE L GL S K QK K KYLGY D W Q I
HIV1 248 ekdswtvndiqklvgklnwasqiypgik--–vrqlckllrgtkal----teviplt-eeaelelae-nreilkepvhgv-yydpskd 324
MLV 291 qptpktprqlreflgtagfc-rlwipgfaEmaaply-pltkt-gtlfnwgpdqqkay--qeikqall-tapalglpdltkpfe---- 367
MMTV 255 lr---tlndfqkllgninwI-rpflklttgelkplfeilngdsnpisirkltpeac-kalqlvn-erlstarvkrldltqpwslcil 335
 T ND QKLLG NW R L L G AE DL P
K70
Y115
dNTP binding sites
Q151
D117
Q155
F119
F120
YxDD motif
B
### Chart
| Category | |
|---|---|
| wt | 100.0 |
| F119V | 0.0 |
| F119L | 0.07142857142857142 |
| F119W | 0.14285714285714285 |
| F119P | 0.07142857142857142 |
| F119Y | 0.14285714285714285 |
| F120L | 15.0 |
| F120W | 0.07142857142857142 |
| F120P | 0.07142857142857142 |
| F120Y | 0.07142857142857142 |
| F120A | 0.07142857142857142 |
| D117A | 0.0 |
| D117E | 0.0 |
| Q155N | 0.07142857142857142 |
| K70R | 0.07142857142857142 |S4 Fig
