# Supplementary material for: A high rate of polymerization during synthesis of mouse mammary tumor virus DNA alleviates hypermutation by APOBEC3 proteins
Source: PLoS Pathog. 2019 Feb 15;15(2):e1007533. doi: 10.1371/journal.ppat.1007533 (PMC6395001; doi:10.1371/journal.ppat.1007533)
Supplement: S5 Fig — The analysis was performed using the HYPERMUT 2.0 program. 2500 (2215 seq. for mA3/F120L) randomly selected sequences were used for analysis. All possible G-to-A changes in the context of the WPRE sequence present in both the MMTV WT and MMTV F120L viruses are shown with the dinucleotide context color. The table below each graph depicts the percent of MMTV sequences carrying the indicated number of mutations within the 270 bp sequence. (PPTX) [file ppat.1007533.s005.pptx]

## Slide 1
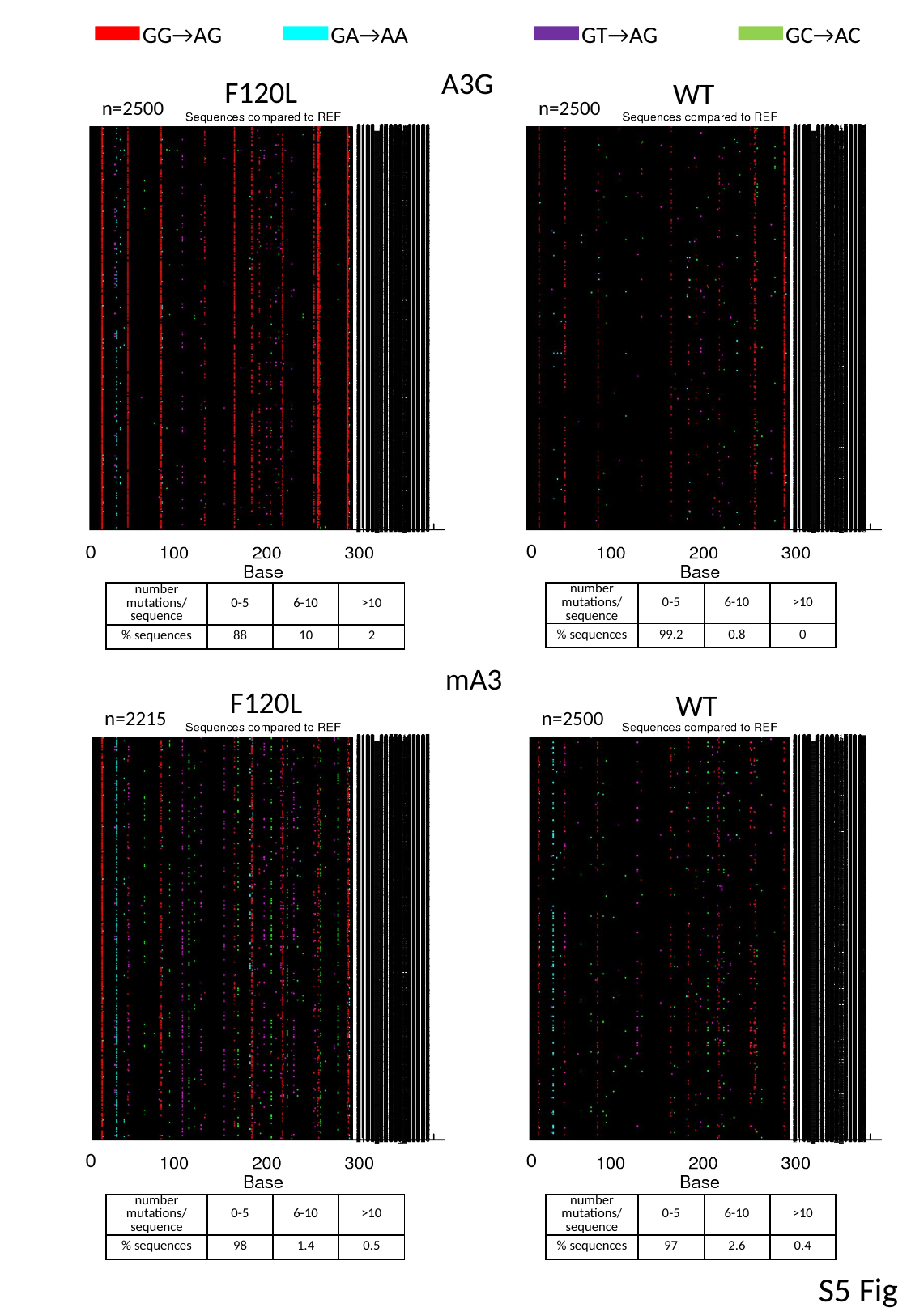

GG→AG
GA→AA
GT→AG
GC→AC
A3G
F120L
n=2500
0
WT
n=2500
0
| number mutations/ sequence | 0-5 | 6-10 | >10 |
| --- | --- | --- | --- |
| % sequences | 88 | 10 | 2 |
| number mutations/ sequence | 0-5 | 6-10 | >10 |
| --- | --- | --- | --- |
| % sequences | 99.2 | 0.8 | 0 |
mA3
F120L
WT
n=2500
0
n=2215
0
| number mutations/ sequence | 0-5 | 6-10 | >10 |
| --- | --- | --- | --- |
| % sequences | 98 | 1.4 | 0.5 |
| number mutations/ sequence | 0-5 | 6-10 | >10 |
| --- | --- | --- | --- |
| % sequences | 97 | 2.6 | 0.4 |
S5 Fig
