# Supplementary material for: A high rate of polymerization during synthesis of mouse mammary tumor virus DNA alleviates hypermutation by APOBEC3 proteins
Source: PLoS Pathog. 2019 Feb 15;15(2):e1007533. doi: 10.1371/journal.ppat.1007533 (PMC6395001; doi:10.1371/journal.ppat.1007533)
Supplement: S6 Fig — (A) Naïve 293T cells were seeded in a six-well plate at a concentration of 3 x 10^5^ cells per well. HU was added next day four hours prior to dNTPs extraction at a final concentration of 0.2 mM. Next, the HU- and mock-treated cells were counted and 0.6 x 10^6^ cells were used for dNTPs extraction followed by dATP quantification as described in Methods. The upper panel shows a schematic diagram of the fluorescence-based assay for the quantification of dATPs. A primer (red arrow) is extended in the presence of dTTP, dCTP and dGTP and the cellular dNTP extract (containing dATP to be measured). When Taq polymerase reaches a probe (green line), its exonuclease activity releases the 6-FAM-labelled nucleotide from the probe resulting in an increase of fluorescence signal. When the dATP from cellular extract becomes exhausted primer extension is terminated leading to no further increase of the fluorescence intensity due to a quenching of fluorescence in the intact probe. The lower panel shows the result of two independent measurements as values normalized to 100% (ctrl = HU-untreated sample). (B) The EGFP expression is delayed in cells treated with hydroxyurea (HU). Cells treated with HU (0.2 mM), when compared to mock-treated cells, show morphological changes including enlarged cell body and nucleus size (see phase contrast figures; 20 hpi). They also prolong their doubling time resulting in a reduced number of cells per well (bottom figures). The onset of EGFP expression was delayed in the HU-treated cells infected with the MMTV-eGFP virus. Whereas the EGFP expression could be detected in mock-treated cells already 24 hpi, the EGFP signal was detectable in HU-treated cells only at later time points (48 hpi). (PPTX) [file ppat.1007533.s006.pptx]

## Slide 1
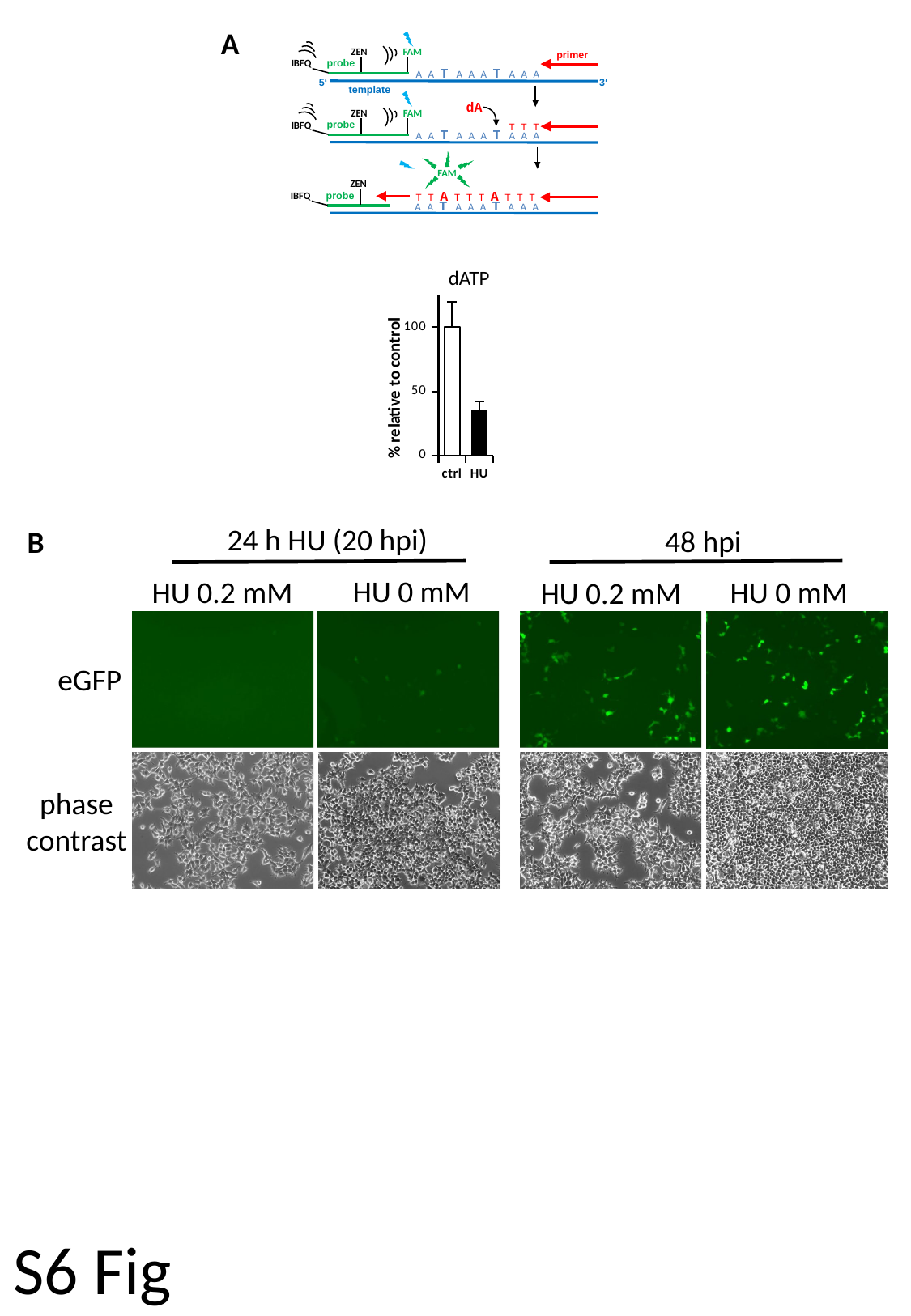

A
ZEN
FAM
primer
probe
IBFQ
A A T A A A T A A A
5‘
3‘
template
dA
ZEN
FAM
 T T T
probe
IBFQ
A A T A A A T A A A
FAM
ZEN
 T T A T T T A T T T
probe
IBFQ
A A T A A A T A A A
dATP
### Chart
| Category | |
|---|---|
| ctrl | 100.0 |
| HU | 35.0 |24 h HU (20 hpi)
48 hpi
HU 0 mM
HU 0.2 mM
HU 0 mM
HU 0.2 mM
eGFP
phase
contrast
B
S6 Fig
