# Supplementary material for: A high rate of polymerization during synthesis of mouse mammary tumor virus DNA alleviates hypermutation by APOBEC3 proteins
Source: PLoS Pathog. 2019 Feb 15;15(2):e1007533. doi: 10.1371/journal.ppat.1007533 (PMC6395001; doi:10.1371/journal.ppat.1007533)
Supplement: S7 Fig — (PPTX) [file ppat.1007533.s007.pptx]

## Slide 1
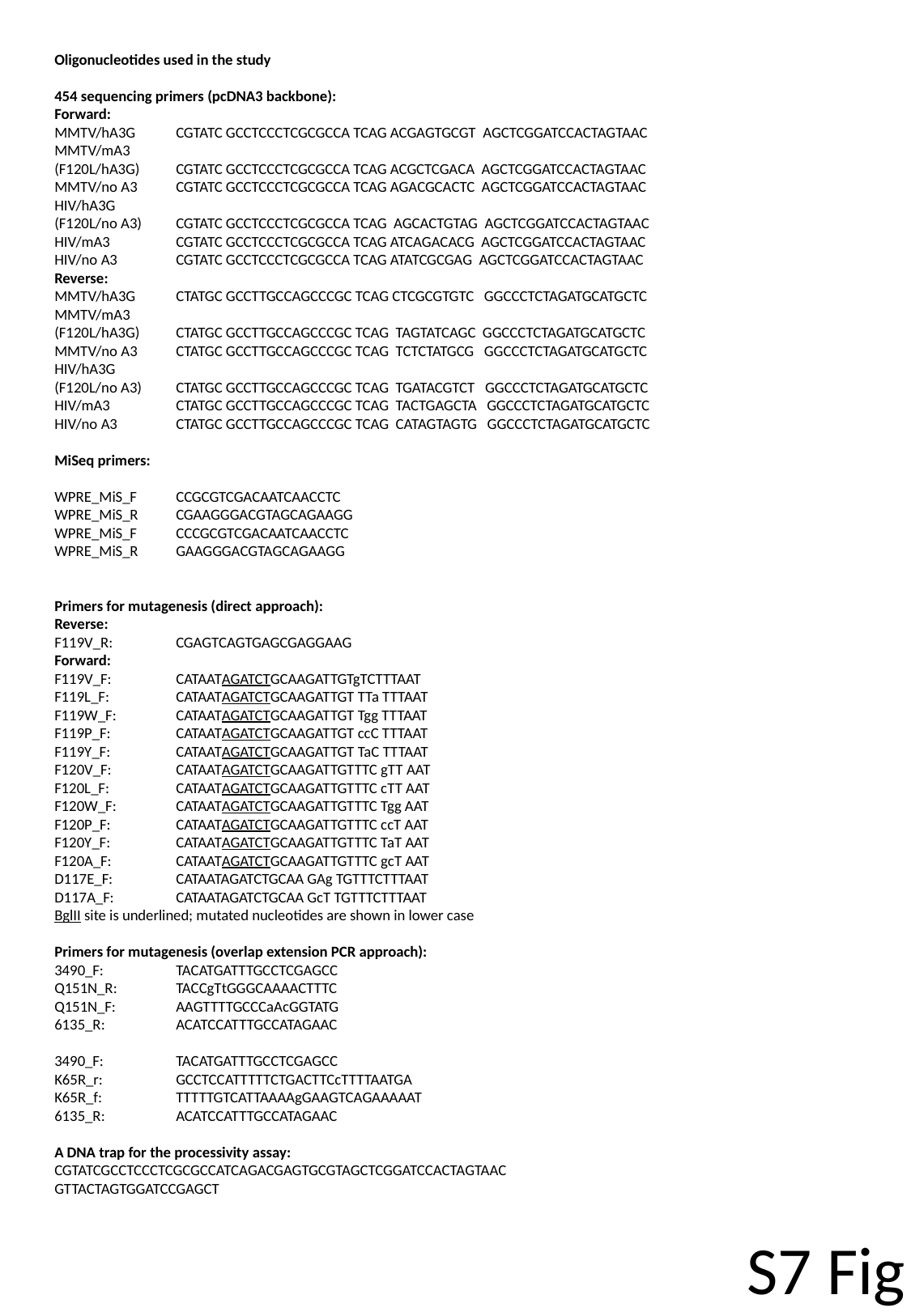

Oligonucleotides used in the study
454 sequencing primers (pcDNA3 backbone):
Forward:
MMTV/hA3G 	CGTATC GCCTCCCTCGCGCCA TCAG ACGAGTGCGT AGCTCGGATCCACTAGTAAC
MMTV/mA3
(F120L/hA3G)	CGTATC GCCTCCCTCGCGCCA TCAG ACGCTCGACA AGCTCGGATCCACTAGTAAC
MMTV/no A3 	CGTATC GCCTCCCTCGCGCCA TCAG AGACGCACTC AGCTCGGATCCACTAGTAAC
HIV/hA3G
(F120L/no A3) 	CGTATC GCCTCCCTCGCGCCA TCAG AGCACTGTAG AGCTCGGATCCACTAGTAAC
HIV/mA3 	CGTATC GCCTCCCTCGCGCCA TCAG ATCAGACACG AGCTCGGATCCACTAGTAAC
HIV/no A3 	CGTATC GCCTCCCTCGCGCCA TCAG ATATCGCGAG AGCTCGGATCCACTAGTAAC
Reverse:
MMTV/hA3G 	CTATGC GCCTTGCCAGCCCGC TCAG CTCGCGTGTC GGCCCTCTAGATGCATGCTC
MMTV/mA3
(F120L/hA3G) 	CTATGC GCCTTGCCAGCCCGC TCAG TAGTATCAGC GGCCCTCTAGATGCATGCTC
MMTV/no A3 	CTATGC GCCTTGCCAGCCCGC TCAG TCTCTATGCG GGCCCTCTAGATGCATGCTC
HIV/hA3G
(F120L/no A3) 	CTATGC GCCTTGCCAGCCCGC TCAG TGATACGTCT GGCCCTCTAGATGCATGCTC
HIV/mA3 	CTATGC GCCTTGCCAGCCCGC TCAG TACTGAGCTA GGCCCTCTAGATGCATGCTC
HIV/no A3 	CTATGC GCCTTGCCAGCCCGC TCAG CATAGTAGTG GGCCCTCTAGATGCATGCTC
MiSeq primers:
WPRE_MiS_F 	CCGCGTCGACAATCAACCTC
WPRE_MiS_R 	CGAAGGGACGTAGCAGAAGG
WPRE_MiS_F 	CCCGCGTCGACAATCAACCTC
WPRE_MiS_R 	GAAGGGACGTAGCAGAAGG
Primers for mutagenesis (direct approach):
Reverse:
F119V_R: 	CGAGTCAGTGAGCGAGGAAG
Forward:
F119V_F: 	CATAATAGATCTGCAAGATTGTgTCTTTAAT
F119L_F:	CATAATAGATCTGCAAGATTGT TTa TTTAAT
F119W_F:	CATAATAGATCTGCAAGATTGT Tgg TTTAAT
F119P_F:	CATAATAGATCTGCAAGATTGT ccC TTTAAT
F119Y_F:	CATAATAGATCTGCAAGATTGT TaC TTTAAT
F120V_F:	CATAATAGATCTGCAAGATTGTTTC gTT AAT
F120L_F:	CATAATAGATCTGCAAGATTGTTTC cTT AAT
F120W_F:	CATAATAGATCTGCAAGATTGTTTC Tgg AAT
F120P_F:	CATAATAGATCTGCAAGATTGTTTC ccT AAT
F120Y_F:	CATAATAGATCTGCAAGATTGTTTC TaT AAT
F120A_F:	CATAATAGATCTGCAAGATTGTTTC gcT AAT
D117E_F:	CATAATAGATCTGCAA GAg TGTTTCTTTAAT
D117A_F:	CATAATAGATCTGCAA GcT TGTTTCTTTAAT
BglII site is underlined; mutated nucleotides are shown in lower case
Primers for mutagenesis (overlap extension PCR approach):
3490_F: 	TACATGATTTGCCTCGAGCC
Q151N_R:	TACCgTtGGGCAAAACTTTC
Q151N_F: 	AAGTTTTGCCCaAcGGTATG
6135_R:	ACATCCATTTGCCATAGAAC
3490_F: 	TACATGATTTGCCTCGAGCC
K65R_r: 	GCCTCCATTTTTCTGACTTCcTTTTAATGA
K65R_f: 	TTTTTGTCATTAAAAgGAAGTCAGAAAAAT
6135_R:	ACATCCATTTGCCATAGAAC
A DNA trap for the processivity assay:
CGTATCGCCTCCCTCGCGCCATCAGACGAGTGCGTAGCTCGGATCCACTAGTAAC
GTTACTAGTGGATCCGAGCT
S7 Fig
